# The development and metamorphosis of the indirect developing acorn worm *Schizocardium californicum* (Enteropneusta: Spengelidae)

**DOI:** 10.1186/s12983-018-0270-0

**Published:** 2018-06-20

**Authors:** Paul Gonzalez, Jeffrey Z. Jiang, Christopher J. Lowe

**Affiliations:** 1 Hopkins Marine Station, Department of Biology, Stanford University, 120 Ocean View Boulevard, Pacific Grove, CA 93950 USA; 2 Department of Chemistry, University of Pennsylvania, 231 South 34th Street, Philadelphia, PA 19104 USA

**Keywords:** Enteropneusta, Hemichordata, Indirect development, Metamorphosis, Planktotrophy, *Schizocardium californicum*, Tornaria

## Abstract

**Background:**

Enteropneusts are benthic marine invertebrates that belong to the deuterostome phylum Hemichordata. The two main clades of enteropneusts are defined by differences in early life history strategies. In the Spengelidae and Ptychoderidae, development is indirect via a planktotrophic tornaria larva. In contrast, development in the Harrimanidae is direct without an intervening larval life history stage. Most molecular studies in the development and evolution of the enteropneust adult body plan have been carried out in the harrimanid *Saccoglossus kowalevskii*. In order to compare these two developmental strategies, we have selected the spengelid enteropneust *Schizocardium californicum* as a suitable indirect developing species for molecular developmental studies. Here we describe the methods for adult collecting, spawning and larval rearing in *Schizocardium californicum*, and describe embryogenesis, larval development, and metamorphosis, using light microscopy, immunocytochemistry and confocal microscopy.

**Results:**

Adult reproductive individuals can be collected intertidally and almost year-round. Spawning can be triggered by heat shock and large numbers of larvae can be reared through metamorphosis under laboratory conditions. Gastrulation begins at 17 h post-fertilization (hpf) and embryos hatch at 26 hpf as ciliated gastrulae. At 3 days post-fertilization (dpf), the tornaria has a circumoral ciliary band, mouth, tripartite digestive tract, protocoel, larval muscles and a simple serotonergic nervous system. The telotroch develops at 5 dpf. In the course of 60 days, the serotonergic nervous system becomes more elaborate, the posterior coeloms develop, and the length of the circumoral ciliary band increases. At the end of the larval stage, larval muscles disappear, gill slits form, and adult muscles develop. Metamorphosis occurs spontaneously when the larva reaches its maximal size (ca. 3 mm), and involves loss and reorganization of larval structures (muscles, nervous system, digestive tract), as well as development of adult structures (adult muscles, tripartite body organization).

**Conclusions:**

This study will enable future research in *S. californicum* to address long standing questions related to the evolution of axial patterning mechanisms, germ layer induction, neurogenesis and neural patterning, the mechanisms of metamorphosis, the relationships between larval and adult body plans, and the evolution of metazoan larval forms.

## Background

Hemichordates (enteropneusts and pterobranchs) are one of the three deuterostome phyla. Together with echinoderms, they form Ambulacraria, the sister-group to chordates [1–8]. Because of their phylogenetic position, hemichordates are crucial to our understanding of early deuterostome evolution and the origins of chordates [9]. Hemichordates include two classes, Enteropneusta and Pterobranchia. Most developmental studies have focused on enteropneusts, or acorn worms. Enteropneusts are marine benthic worms with a tripartite body plan, divided along the anteroposterior (AP) axis into a preoral proboscis, a collar that encloses the buccal cavity, and a long trunk that comprises an anterior pharynx perforated by gill slits, the digestive tract and gonads. In enteropneusts, this adult body plan can develop through two radically different developmental strategies, direct and indirect development [6, 10, 11]. This diversity of life history strategies provides both challenges and opportunities for comparative developmental studies within the phylum, and with other metazoan phyla.

In the enteropneust family Harrimanidae, all known species are direct developers. Females spawn a small number (between a hundred and a thousand) of large yolky eggs [12, 13] that range between 230 μm and 1.3 mm in diameter [14]. In this group, development progresses directly towards formation of a benthic juvenile that resembles a miniature version of the adult [11]. Embryonic development begins within the fertilization envelope and hatching can occur anywhere between gastrulation and formation of the juvenile [12, 14, 15]. Hatching may be followed by a brief non-feeding pelagic stage, in which the developing acorn worm swims using a band of cilia called the telotroch, before settling on the benthos. The adult body plan with a proboscis, collar and trunk is typically visible within a few days after fertilization. The harrimanid *Saccoglossus kowalevskii* was developed as a research organism for molecular developmental studies [13, 16], and comparisons with chordates have provided genetic and developmental insights into the evolution of deuterostome body plans [9, 10].

The other three enteropneust families, Ptychoderidae, Spengelidae and Torquaratoridae form a monophyletic sister-clade to the Harrimanidae [17]. Early development is unknown in the Torquaratoridae, but all ptychoderid and spengelid species for which development is described are indirect developers [11]. Unlike harrimanids, indirect developing enteropneusts spawn large numbers of small eggs that range between 60 and 160 μm [14]. The embryo typically hatches shortly after gastrulation, and develops into a planktotrophic tornaria larva that has a distinct body plan from the adult. Tornaria larvae are small (1–9 mm) [14], transparent and morphologically simple. They swim using a telotroch similar to direct developing species, but feed by capturing unicellular algae using specialized bands of cilia. Their body cavity is made up of the embryonic blastocoel and filled with a gel-like material [18]. They have a simple digestive tract and an anterior sensory organ called the apical organ. After spending weeks to months in the plankton the tornaria metamorphoses into a benthic juvenile worm with a proboscis, collar and trunk.

Most of what we know about indirect development in enteropneusts comes from studies in ptychoderids. Most early descriptions of tornaria larvae were made on individuals collected from the plankton, and lack descriptions of embryonic development, metamorphosis into the benthic juvenile, and/or identification of the corresponding adult species. The first detailed descriptions of the tornaria were made in the late nineteenth century on ptychoderids from the genus *Balanoglossus* [19, 20]. Recent studies have mostly focused on *Ptychodera flava*, and include descriptions of embryonic development, larval morphology, neuroanatomy, feeding mechanisms, and general aspects of metamorphosis [21–26]. Recent developmental descriptions were also made in *Balanoglossus simodensis* [27, 28] and *Balanoglossus misakiensis* [29, 30]. Larval development was described in only two Spengelid species, both from the genus *Glandiceps* [31, 32]. Among indirect developing hemichordates, *Ptychodera flava* has been used as the main research organism for molecular developmental studies, and more recently *Balanoglossus misakiensis* and *Balanoglossus simodiensis* have been added to the list [16]. All three species belong to the Ptychoderidae.

Cross phyla comparisons of developmental mechanisms generally compare embryogenesis between distantly related species with little consideration of the equivalence of the body plan that arises directly from the embryo (larval, or adult). Molecular developmental studies within phyla comparing developmental strategies between animals with similar adult body plan, but contrasting developmental strategies are rare (but see [33, 34] for such comparisons in nemerteans and [35–47] for comparisons in sea urchins). However, these comparisons are essential to determine which developmental mechanisms are broadly shared, representing conserved traits within a phylum, and which ones are associated with a specific life history strategy. Additionally, this type of comparative data set is important to address outstanding zoological questions concerning the origins and evolutionary history of metazoan larval forms. Molecular developmental studies in hemichordates are ideal to address these questions as the two main enteropneust lineages have strongly contrasting developmental modes.

We established an enteropneust from California, *Schizocardium californicum* as a new species representing indirect development amenable to molecular developmental studies. Current indirect developing models are all distributed in the tropical and subtropical waters of the Indo-Pacific. Therefore, the addition of *Schizocardium californicum* expands the geographical range of indirect developing species available for molecular work. Additionally, *Schizocardium californicum* is a member of Spengelidae, a family that was not previously represented in hemichordate research organisms, and can be easily collected intertidally.

Here, we provide a detailed description of embryogenesis, larval development, and metamorphosis in *Schizocardium californicum*, as well as the methods for adult collecting, spawning and larval rearing. This work not only provides a useful resource for future work in *Schizocardium californicum*, but also expands our knowledge of the diversity of development, larval morphology and metamorphosis in enteropneusts.

## Methods

### Animal collection

Our collection site is in Morro Bay State Park, California, in a mudflat located at 35°20′56.7”N 120°50′35.6”W. Water temperature in Morro Bay varies seasonally between 11 °C and 16 °C (https://www.seatemperature.org). Adult *Schizocardium californicum* are found in the lower intertidal and upper subtidal zone throughout the mudflat. *Schizocardium californicum* was reported from this locality as *Schizocardium sp*. in two studies [48, 49], and the species was later formally described from specimens collected at another locality in southern California [50].

Unless at exceptionally low tides, collecting must be done at a depth of ca. 1 m for best results. Worms are usually found where the sediments form a mixture of sand and organic matter. They are not found in the sandy upper intertidal zone. Worms can be collected at low tide using a shovel. After bringing sediments to the surface, we pull the mud apart to expose the burrows and manually pull the worms out. The type of sediment is not suitable for using a sieve. Worms are placed in individual 50 mL Falcon tubes with sea water and kept in a cooler containing ice during transportation to prevent heat-induced spawning. Worms should be kept in flowing seawater tables in individual finger bowls with enough mud to cover them, and the sea tables must be kept in the dark to help prevent spontaneous spawning.

Adult worms used in this study were collected at regular intervals between February 2014 and January 2017 and transported to Hopkins Marine Station.

### Spawning

Glassware used for spawning, fertilization and larval rearing must never come in contact with detergents or fixatives. To induce spawning, females are removed from the mud, transferred to individual bowls of sea water filtered using a 0.2 μm mesh, and placed in an illuminated incubator at 24–26 °C. Animals are monitored every 20 min during incubation. Spawning can occur anywhere from 20 min to 12 h after initiation of heat shock. Generally, between a third and a half of the collected females can be induced to spawn. After spawning, eggs are transferred to a bowl of 14 °C filtered sea water (FSW) using a plastic pipette with a large opening (strong water flow may cause egg activation and should be avoided). From this point on, eggs and embryos must be kept at 14 °C and temperature variation should be kept to a minimum. We typically spawn females no later than three days after collection, as gravid females often spawn spontaneously in the sea tables, and most of them are spawned out after a few days. Keeping females in mud and in the dark may reduce spontaneous spawning.

To collect sperm, the epidermis of the trunk in the genital region is ruptured using forceps or small scissors, causing large quantities of sperm to be released in ripe individuals. Sperm can be collected with a Pasteur pipette and kept on ice. Alternatively, heat shock similar to female treatment can trigger release of sperm. An average size male can release several milliliters of sperm at once. There is considerable variation in sperm quality between males, and generally several males need to be checked for sperm motility before active sperm is found. After dissection, sperm is viable for at least 8 h.

### Fertilization

Eggs are fertilized using methods adapted from studies in echinoderms [51] and other hemichordates [13]. One drop of concentrated sperm is diluted in 10 mL of FSW, and 1 mL of this solution is added to a small bowl containing eggs in ~ 200 mL FSW. After gently stirring the water, the eggs are monitored under a dissecting microscope. A fertilization envelope becomes visible around the oocytes that have been successfully fertilized 4 to 5 min after addition of sperm. If the fertilization rate is lower than 90–95%, additional sperm solution may be added until most eggs are fertilized. Fertilized eggs are transferred to a large dish with flat bottom and remaining sperm is eliminated by changing the water three times. Fertilized oocytes must form a single layer at the bottom of the dish.

### Larval rearing

At 1 dpf, hatched embryos begin to swim and are phototactic. At this point, they can be easily separated from any unfertilized oocytes that may remain at the bottom of the dish, and are transferred to 1 gal glass jars. The water must be stirred continuously and kept at 14 °C. Feeding begins immediately after mouth formation, at 3 dpf. We feed larvae with a 1:1 mix of *Dunaliella tertiolecta* and *Rhodomonas lens*, but they can survive and grow on a pure *D. tertiolecta* diet. Algae are reared as outlined in [52]. The volume of algae solution needed depends on algal culture density and larval density. We typically start with 200 mL of high density algae solution per jar. We later adjust this amount so that algal cells can be seen in the stomach of all larvae at all time. Larvae are examined every day and algae is added to the water if more than ~ 20% of the larvae have empty stomachs. Every three days, water is replaced with clean FSW, containers are washed, and fresh algae is added. At early larval stages, larvae are kept at a density of 1 larva/mL. As larvae grow the density needs to be decreased progressively. When tornaria larvae reach 2 mm in size, they are kept at a density of around 50 individuals per liter.

### Light microscopy

Live larvae were immobilized on slides coated with poly-L-lysine and imaged using a Zeiss Imager Z1 microscope using DIC optics. Larvae that were larger than the field of view at 5X (after 50 dpf) were photographed in two or three partial pictures that were later merged using the Adobe Photoshop CS6 Photomerge function. Metamorphosing larvae and juveniles were relaxed in a 1:1 mix of 7.5% MgCl_2_ and FSW prior to imaging.

### Immunolabeling and laser scanning confocal microscopy

Samples were fixed in 3.7% formaldehyde in fixation buffer (1X phosphate buffered saline (PBS), 0.1 M MOPS, 0.5 M NaCl, 2 mM EGTA, 1 mM MgCl_2_) for 1 h at room temperature (RT), and washed 3 times (5 min each) in fixation buffer (based on [13]). After formation of the apical strand (5 dpf and older), larvae were relaxed in a 1:1 mix of 7.5% MgCl_2_ and FSW for 5 min before fixation to prevent retraction of the apical plate. Samples were washed 3 times (5 min each) in PBT (PBS + 0.1% TritonX-100 + 0.1% bovine serum albumin) and blocked in PBT + 5% goat serum for 1 h at RT. Antibodies were then added directly to the blocking solution. To visualize cilia, we used a mouse monoclonal anti-acetylated tubulin antibody (Sigma T7451) diluted 1:400 in blocking solution. To visualize the serotonergic nervous system, we used a rabbit anti-serotonin antibody (Sigma S5545) diluted 1:300 in blocking solution. Samples were incubated in antibody solution overnight at 4 °C. Samples were then washed 3 times for 1 min each and 3 times for 20 min each in PBT and blocked as previously. Secondary antibodies (ThermoFisher, Alexa Fluor goat anti-mouse IgG 555 (A-32727) and Alexa Fluor goat anti-rabbit IgG 633 (A-21070)) and BODIPY FL-phallacidin (ThermoFisher B607) were added at 1:1000 dilution to the blocking solution. Samples were then washed in PBT 3 times for 1 min each and 3 times for 20 min each. Samples were stained with DAPI (1 μg/mL in PBS), and mounted in PBS using coverslips elevated with clay feet, and sealed with nail polish. Glycerol was not used in order to prevent deformation of natural tissue shape. Samples were imaged on a Zeiss LSM 700 with 10X, 20X and 40X objectives. For samples larger than the field of view, maximal intensity projections from several stacks were stitched together using the Tile tool (Zen2 software, Zeiss) followed by manual alignment of tiles in Photoshop when necessary.

## Results

### Spawning and fertilization

**Fig. 1:**
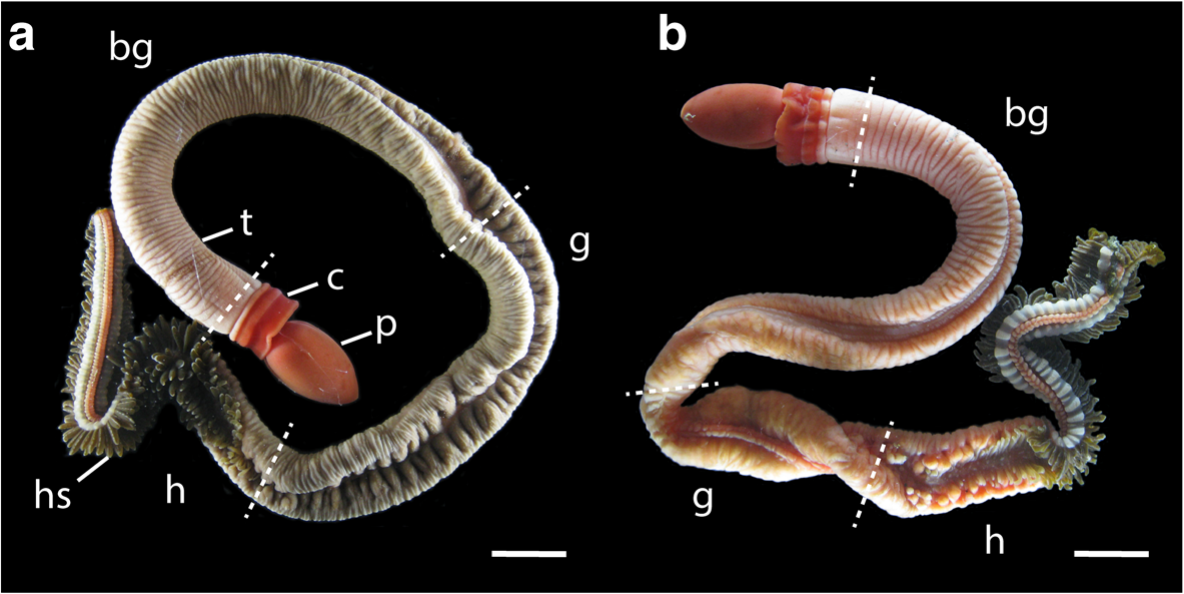
Adult *Schizocardium californicum*. Light micrographs. **a** Female. **b** Male**.** Dashed lines mark the boundaries between the main body regions. bg, branchiogenital region; c, collar; g, genital region; h, hepatic region; hs, hepatic sacs; p, proboscis; t, trunk. Scale bars: 10 mm

Adult *Schizocardium californicum* measure up to 30 cm in length. Gravid females are darker in color than males (Fig. 1). Gonads are present throughout the length of the branchiogenital and genital regions of the trunk (Fig. 1). Most individuals contain gametes year-round. Although we observe a decrease in egg and sperm quality during July and August, we are able to routinely spawn adults and obtain embryos from September to June.

**Fig. 2:**
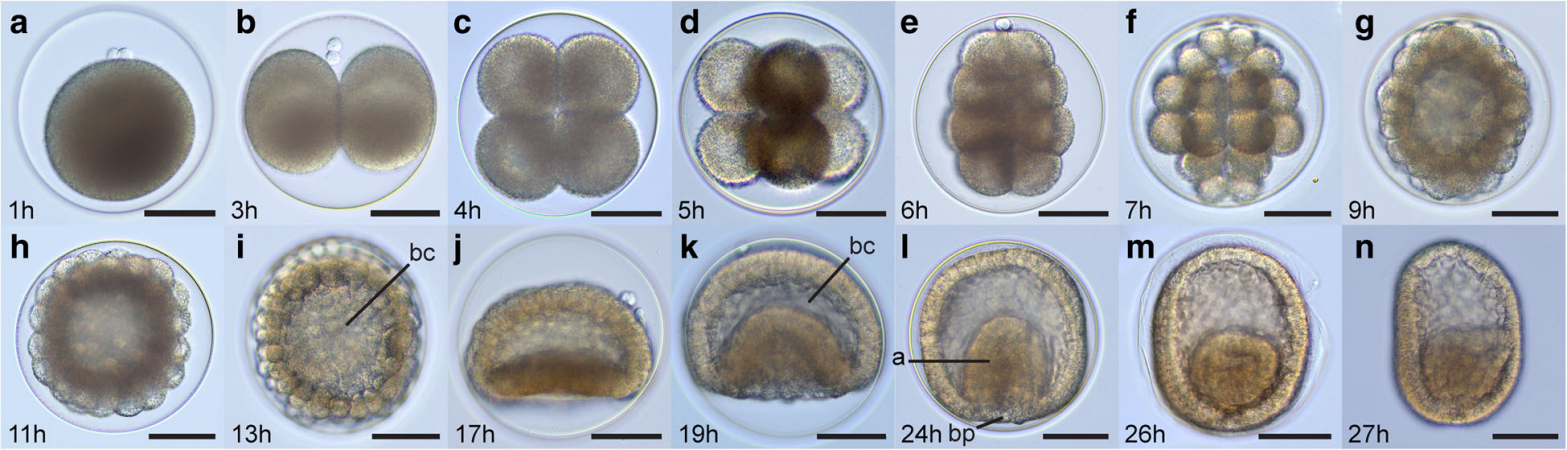
Cleavage and gastrulation in *Schizocardium californicum*. Light micrographs. Animal pole is up. **a** Zygote. **b** 2-cell stage. **c** 4-cell stage. **d** 8-cell stage. **e** 16-cell stage. **f** 32-cell stage. **g**-**i** Formation of the coeloblastula. **j** Flat plate gastrula. **k** Mid-gastrula. **l** Pre-hatching gastrula. **m** Gastrula at time of hatching. **n** Post-hatching gastrula. a, archenteron; bc, blastocoel; bp, blastopore. Scale bars: 50 μm

Hundreds of thousands of eggs may be released in a single spawning event. Eggs are released through pores located between pseudosegmental folds of the trunk epidermis. Eggs are pink/purple and measure approximately 110 μm in diameter. After spawning and until fertilization, the germinal vesicle is visible as a spot of lighter color. At fertilization, the fertilization envelope forms and raises around the zygote. After about an hour, the germinal vesicle disappears and the polar bodies are visible at the animal pole (Fig. 2a).

### Embryonic development

At 14 °C, first cleavage occurs at 3 hpf and subsequent cleavages occur at intervals of ca. 1 h. Cleavage is holoblastic and radial as in other enteropneusts [53]. The first two cleavages occur along the animal-vegetal (A/V) axis, and are equal and orthogonal to each other (Fig. 2b-c). The third cleavage is equatorial and equal and results in the formation of two tiers of 4 blastomeres sitting on top of each other (Fig. 2d). Fourth cleavage is equatorial and unequal, resulting in a 16-cell embryo made of 4 tiers of 4 blastomeres where animal and vegetal tiers of cells are smaller than the central two tiers (Fig. 2e). Subsequently, the two sets of central blastomeres cleave along the A/V axis, resulting in two central sets of 8 blastomeres of equal size, while unequal cleavage in the animal- and vegetal-most cells results in smaller blastomeres at the animal and vegetal poles at the 32-cell stage (Fig. 2f).

Over the following hours, the embryo becomes a coeloblastula made up of a few hundred cells (Fig. 2g-i). At 17 hpf, gastrulation begins with the flattening of the vegetal side of the embryo (Fig. 2j), which becomes its posterior pole. The presumptive endomesoderm invaginates as cells continue to divide (Fig. 2k), and the blastopore decreases in size (Fig. 2l). The gastrula is ciliated and begins to rotate within the fertilization envelope. At 26 hpf, the fertilization envelope ruptures, and the gastrula hatches out and swims (Fig. 2m). At the time of hatching, the roof of the archenteron flattens and extends towards the dorsal side of the embryo. The embryo elongates along the AP axis (Fig. 2n), and measures ca. 150 μm in length 1 h after hatching.

### Early larval development

The developmental events described below are summarized in Table 1, together with the corresponding “traditional” staging terminology of enteropneust larval and metamorphic stages. The terminology concerning ciliary bands, fields and grooves of the larval ectoderm is not entirely consistent across existing descriptions of tornaria morphology. Here, we use the terminology described in the widely used monograph by van der Horst [54] and based on earlier work by Stiasny-Winjhoff and Stiasny [55, 56]. We mention alternative terms from [24] when relevant.

**Table 1:** Summary and timeline of morphological changes during development and metamorphosis of *Schizocardium californicum.* Traditional staging terminology is copied from [14]

Time	Ectoderm	Mesoderm	Endoderm	Traditional staging terminology
3–5 dpf	Circumoral ciliary band 2–4 serotonergic neurons in apical organ Apical tuft	Apical strand Pharyngeal circular muscles Protocoel, pore canal and hydropore	Tripartite gut	Müller stage Larva just recognizable as a tornaria, longitudinal ciliary bands formed, telotroch not yet present
5–10 dpf	Eyespots Telotroch formation, neurotroch Neuropil, dorsal axons	Anchoring muscles		Heider stage Longitudinal ciliary bands without development of lobes and saddles; telotroch just formed.
10–18 dpf	Dorsal cluster of 5HT neurons in apical organ Telotroch fully formed			
18–22 dpf	Primary dorsal saddles and lobes Ventral saddle Addition of cell bodies in apical organ			Metschnikoff stage Primary lobes and saddles in formation or formed. Trunk coeloms not yet or having just appeared.
22–30 dpf	Apical tuft disappears Primary ventral saddles and lobes Lower dorsal lobe	Longitudinal muscles in pharynx Mesocoels and metacoels		Krohn stage Secondary lobes and saddles or tentacles formed or forming. Trunk coelom usually present. Collar coelom not yet present or just first appearing. Large size. The high point of larval development.
30–50 dpf	Secondary telotroch	Pulsatile vesicle		
50–65 dpf	Serotonergic cell bodies throughout ectoderm Well-developed serotonergic nerve net	Apical strand disappears Mesocoels and metacoels increase in size Muscle fibers in coeloms	Gill slits (5 pairs)	Spengel stage Secondary lobes and saddles in regression or regressed. Collar and trunk coeloms present. Smaller than the previous stage, club shaped. Circular constriction about the middle of the body. Opaque.
0–5 h post onset of metamorphosis	Thickening of epidermis Gill pore formation	Expansion of all coeloms (reduction of blastocoelar space)	Digestive tract retracts posteriorly	
5–11 h post-onset of metamorphosis	Food grooves disappear Circumoral ciliary band disappears	Protocoel replaces blastocoelar space in proboscis Larval pharyngeal muscles disappear Trunk musculature develops	Pharynx posterior to the mouth	Agassiz stage Secondary lobes and saddles gone. Collar and trunk coeloms well developed. Regionalization indistinct. Entire body more elongate than in previous stage. Analfield a conical bulge. Longitudinal ciliary bands shifted toward the apical plate, the gut shifted analward. Protocoel very large.
12–24 h post-onset of metamorphosis	Epidermis opaque Protocoel fills entire proboscis	Mesocoels replace blastocoelar space in collar Well-developed longitudinal muscles in trunk	Growth of pharynx Growth of gill slits Stomach folds	
24–48 h post-onset of metamorphosis	Resorption of telotroch Transition from swimming to burrowing Eyespots disappear	Metacoels replace blastocoelar space in trunk	Additional gill slits	Metamorphosis stage Metamorphosis begins at the end of larval life. Proboscis, collar, and trunk regions delineated. Ciliary rows or tentacles in atrophy. Appearance of gill slits and buccal diverticulum.
	In all germ layers: clear division of body into proboscis, collar and trunk. Trunk growth			

**Fig. 3:**
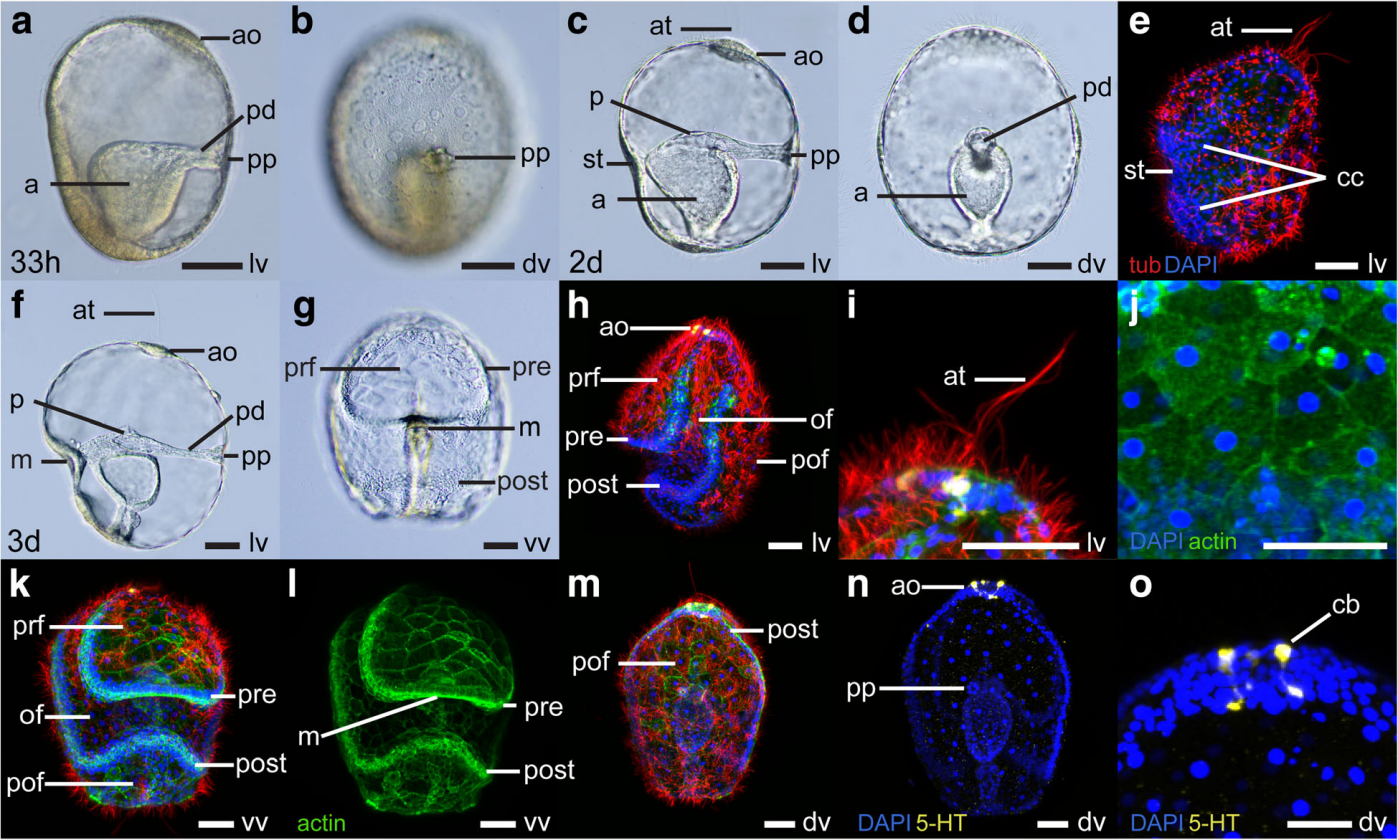
Early larval development in *Schizocardium californicum*. **a**-**d**, **f**-**g** Light DIC micrographs of live specimens. Optical sections through the middle of the embryo (**a**, **c**-**d**, **f**) or surface views (**b**, **g**). **e**, **h**-**o** Maximum intensity projections of confocal stacks. Larvae are labeled with four markers: phalloidin (green), anti-acetylated tubulin (red) and anti-serotonin (yellow) antibodies, and DAPI (blue) unless otherwise indicated. All panels are anterior to the top. Lateral views are ventral to the left. **a**, **b** Hatched gastrula stage embryos at the time of pore canal formation, 33 hpf. **c**-**e** Developing tornaria larva at the time of protocoel formation, 2 dpf. **f**-**o** Early tornaria after mouth formation, 3 dpf. **i** Close-up lateral view of the apical organ_._ **j** Close-up view of the squamous epithelium of the dorsal ectoderm. **o** Close-up dorsal view of the apical organ. a, archenteron; ao, apical organ; at, apical tuft; cb, serotonergic cell body; cc, cells of the presumptive circumoral ciliary band; dv, dorsal view; lv, lateral view; m, mouth; of, oral field; p, protocoel; pd, protocoel duct (pore canal); pof, postoral field; post, postoral loop of the circumoral ciliary band; pp, protocoel pore (hydropore); pre, preoral loop of the circumoral ciliary band; prf, preoral field; st, stomodeum; vv, ventral view. Scale bars: 50 μm

During the first few hours after hatching, the presumptive mesoderm at the roof of the archenteron fuses with the dorsal ectoderm, forming the protocoel pore, (also called hydropore or proboscis pore) connected at this stage to the archenteron via the protocoel duct, or pore canal (Fig. 3a-b). The pore canal-hydropore complex constitutes the larval protonephridial system, an excretory system that uses a cilia-driven flow for ultrafiltration of coelomic fluid from the protocoel [57, 58]. At this stage however the protocoel itself, which forms by enterocoely over the next two days of development, has not yet separated from the archenteron. During this time, the ectoderm becomes thinner and the blastocoelar space enlarges, as the embryo grows to ca. 260 μm at 48 hpf (Fig. 3c-d).

At 48 hpf, the stomodeum forms as a depression in the ventral ectoderm midway along the AP axis (Fig. 3c, e). The anterior part of the archenteron bends towards the stomodeum and makes contact with the ectoderm at this level, beginning the process of mouth formation (Fig. 3c). At the same time, the protocoel begins to pinch off from the archenteron. At this stage, the monociliated cells that will form the future circumoral ciliary band (sometimes called neotroch [24], or longitudinal ciliary band [14]) begin to form regions of higher cell density (Fig. 3e). The developing apical plate is visible as a region of thick columnar epithelium at the anterior dorsal end of the embryo (Fig. 3c). At this stage, cells of the apical plate bear a group of very long cilia called the apical tuft (Fig. 3e), but no serotonin immunoreactivity is detected.

At 3 dpf, when the larva measures ca. 300 μm, the mouth perforates the ectoderm (Fig. 3f-g) at the center of the oral field (the region located between the two loops of the circumoral ciliary band) and the larva begins to feed. At this stage, the ciliary band is clearly distinct from the general ectoderm, and comprises the majority of the cells of the embryo (Fig. 3h, k, m). Cells of the ciliary band are tightly grouped together and create a visible thickening of the epidermis, while the remaining ectoderm is made of very thin wide multiciliated cells forming a squamous epithelium (Fig. 3g-h, j-n). Cells located in the two regions delimited by the presumptive ciliary bands form the preoral and postoral fields (also called frontal aboral field and dorsal aboral field respectively) (Fig. 3h, k, m). These cells are multiciliated and bear cilia that are longer than those of the forming circumoral ciliary band. Locomotion is presumably achieved by these cilia at this stage. The gut is subdivided in three parts, with an anterior pharynx (still reduced at this stage), an intermediate stomach and posterior intestine (Fig. 3f). The larval pharynx is occasionally referred to as oesophagus in the literature, but we prefer the term pharynx, as this structure becomes the pharynx of the adult (described below). The protocoel pinches off from the archenteron at 3 dpf and sits anterior to the stomach (Fig. 3f). Between two and four serotonergic neurons are visible in the apical plate, and together with the apical tuft of cilia, they form the apical organ of the larva (Fig. 3h-i, m-o).

**Fig. 4:**
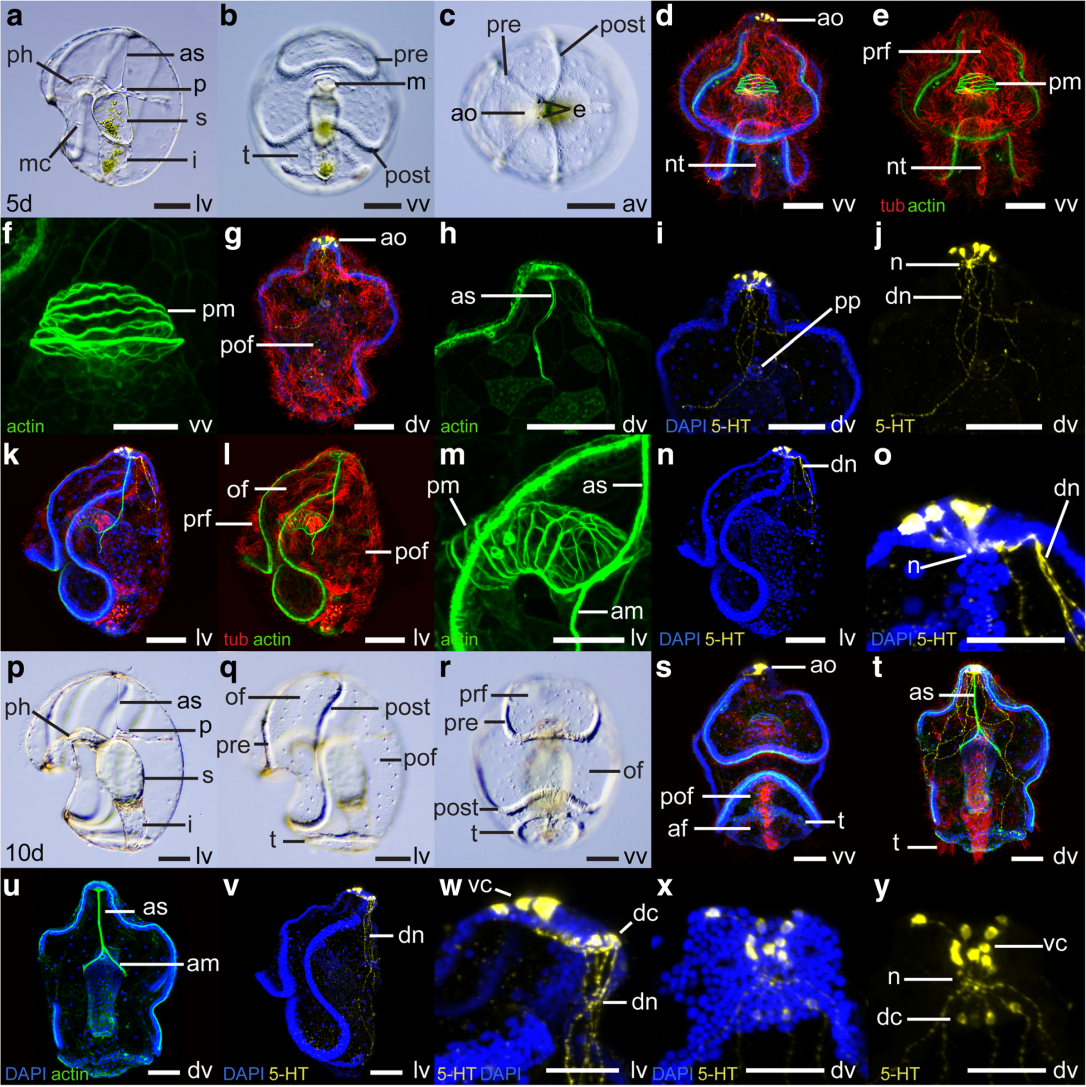
Morphology of the tornaria larva of *Schizocardium californicum* at 5 dpf and 10 dpf. **a**-**c, p**-**r** Light DIC micrographs of live specimens. Optical sections through the middle of the embryo (**a**-**p**) or surface views (**b**-**c**, **q**-**r**). **d**-**o**, **s**-**y** Maximum intensity projections of confocal stacks. Larvae are labeled with four markers: phalloidin (green), anti-acetylated tubulin (red) and anti-serotonin (yellow) antibodies, and DAPI (blue) unless otherwise indicated. All panels are anterior to the top, and lateral views are ventral to the left. **a**-**o** Tornaria larva at 5 dpf. **f**, **m** Close-up views of the circular pharyngeal muscles and apical strand. **i**-**j, o** Close-up views of the apical organ and dorsally projecting neurites. **p**-**y** Tornaria larva at 10 dpf. **w**-**y** Close-up views of the apical organ and dorsal nerve tract. af, anal field; am, anchoring muscles; ao, apical organ; as, apical strand; av, anterior view; dc, dorsal cluster of the apical organ; dn, dorsal nerve tract; dv, dorsal view; e, eyespots; i, intestine; lv, lateral view; m, mouth; mc, mesenchymal cell; n, neuropil; nt, neurotroch; of, oral field; p, protocoel; ph, pharynx; pm, pharyngeal muscles; pof, post-oral field; post, postoral loop of the circumoral ciliary band; pp, protocoel pore (hydropore); pre, preoral loop of the circumoral ciliary band; prf, preoral field; s, stomach; t, telotroch; vc, ventral cluster of the apical organ; vv, ventral view. Scale bars: 100 μm (a-e, g-l, p-v), 50 μm (f, m, o, w-y)

At 5 dpf (Fig. 4a-o), the larva measures ca. 440 μm. Two pigmented eyespots are visible at the surface of the apical plate (they first appear at 4 dpf) (Fig. 4c). The pharynx curves anteriorly before reaching the stomach (Fig. 4a), and as a result the protocoel is located dorsal to the pharynx and anterior to the stomach. A group of circular muscles surrounds the pharynx (Fig. 4d-f, l-m), and a strand of muscle fibers, the apical strand, connects the protocoel to the apical plate (Fig. 4a, h, l-m). The apical strand is anchored to the junction between pharynx and stomach by two anchoring muscle fibers (Fig. 4k-m). All of these larval muscles presumably originate from mesenchyme cells, which become widespread at this stage (Fig. 4a). In the posterior end of the larva, a circular thickening of the ectoderm forms at the level of the future telotroch (Fig. 4b). A band of multiciliated cells, the neurotroch, runs along the ventral midline between the anus and the postoral loop of the circumoral ciliary band (Fig. 4d-e). Additional serotonergic neurons develop in the apical organ (Fig. 4i-j, n-o). Cell bodies are located in the ventral region of the apical plate. Neurites form a neuropil at the base of the apical plate and project to the dorsal ectoderm.

### Later larval development

At 10 dpf (Fig. 4p-y), the larva measures ca. 550 μm and differs from earlier stages mainly by the presence of the telotroch, which develops as a ring of tightly grouped cells bearing long cilia in the posterior ectoderm (Fig. 4p-t). The telotroch is sometimes called opistotroch [24]. The telotroch separates the postoral field from the anal field, the region located between the telotroch and the anus. At 10 dpf, serotonergic cell bodies become apparent in the dorsal region of the apical plate. During the remainder of larval development, the apical organ will be divided between two discrete ventral and dorsal clusters of neurons, connected by a neuropil (Fig. 4v-y).

**Fig. 5:**
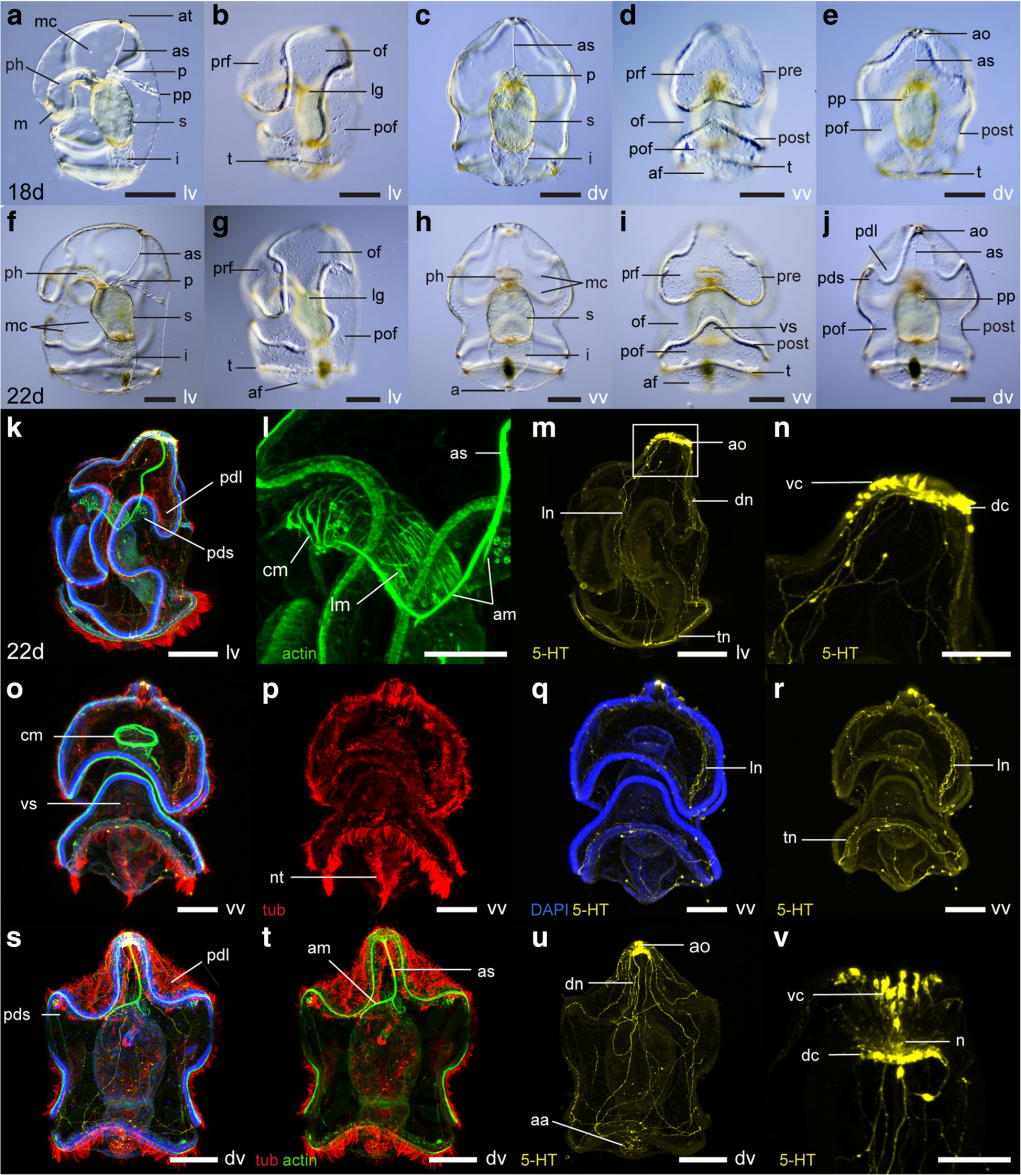
Morphology of the tornaria larva of *Schizocardium californicum* at 18 and 22 dpf. **a**-**j** Light DIC micrographs of live larvae. Optical sections through the middle of the embryo (**a**, **c**, **f**, **h**) or surface views (**b**, **d**-**e**, **g**, **i**-**j**). **k**-**v** Maximum intensity projections of confocal stacks. Larvae are labeled with four markers: phalloidin (green), anti-acetylated tubulin (red) and anti-serotonin (yellow) antibodies, and DAPI (blue) unless otherwise indicated. All panels are anterior to the top, and lateral views are ventral to the left. **a**-**e** Tornaria larva at 18 dpf. **f**-**v** Tornaria larva at 22 dpf. **l** Close-up lateral view of pharyngeal circular and longitudinal muscles. **n** Close-up lateral view of apical organ (box in panel m). **v** Close-up dorsal view of apical organ. a, anus; aa, autofluorescence caused by algal cells in digestive tract; af, anal field; am, anchoring muscles; ao, apical organ; as, apical strand; at, apical tuft; cm, circular pharyngeal muscles; dc, dorsal cluster of the apical organ; dn, dorsal nerve tract; dv, dorsal view; i, intestine; lg, lateral groove; lm, longitudinal pharyngeal muscles; ln, lateral nerve tract; lv, lateral view; m, mouth; mc, mesenchymal cell; n, neuropil; nt, neurotroch; of, oral field; p, protocoel; pdl, primary dorsal lobe; pds, primary dorsal saddle; ph, pharynx; pof, postoral field; post, postoral loop of the circumoral ciliary band; pp, protocoel pore (hydropore); pre, preoral loop of the circumoral ciliary band; prf, preoral field; s, stomach; t, telotroch; tn, telotroch nerve tract; vc, ventral cluster of the apical organ; vs, ventral saddle; vv, ventral view. Scale bars: 200 μm (a-k, m, o-u), 100 μm (l, n, v)

Between 18 dpf (Fig. 5a-e) and 22 dpf (Fig. 5f-v), the larva measures between 800 μm and 1.1 mm. During that time, the shape of the circumoral ciliary band becomes more complex. The ventral part of the postoral loop folds anteriorly, creating the ventral saddle (compare Fig. 5d and i, Fig. 5o-q). Dorsally, the postoral loop folds, creating the primary dorsal saddles (in the postoral field) and the primary dorsal lobes (dorsal extensions of the oral field) (compare Fig. 5e and j, Fig. 5k, s). The lateral regions of the oral field become narrower and are called the lateral grooves (Fig. 5b, g). Cilia of the circumoral ciliary band are noticeably longer (Fig. 5k, o-p, s-t). The apical tuft is still apparent at 18 dpf (Fig. 5a), but disappears by 22 dpf (Fig. 5f, k). At 22 dpf, pharyngeal muscles are divided into anterior circular muscles, and posterior longitudinal muscles (Fig. 5l). Mesenchyme cells become more abundant (Fig. 5a, f, h).

Between 10 and 22 dpf, the serotonergic nervous system becomes progressively more elaborate (Fig. 5m-n, r, u-v). At 22 dpf, the apical organ comprises around 20 serotonergic cell bodies, divided into a ventral and a dorsal cluster, located respectively on the ventral and dorsal sides of the apical plate (Fig. 5m, n, v). These two clusters of neurons project neurites posteriorly, forming lateral neurite bundles under the oral field on each side (Fig. 5m, r), and a dorsal neurite bundle in the postoral field (Fig. 5m, u). A neurite bundle forms under the telotroch (Fig. 5m, r). Neurites from the dorsal and lateral neurite bundles connect to this telotroch bundle (Fig. 5m).

**Fig. 6:**
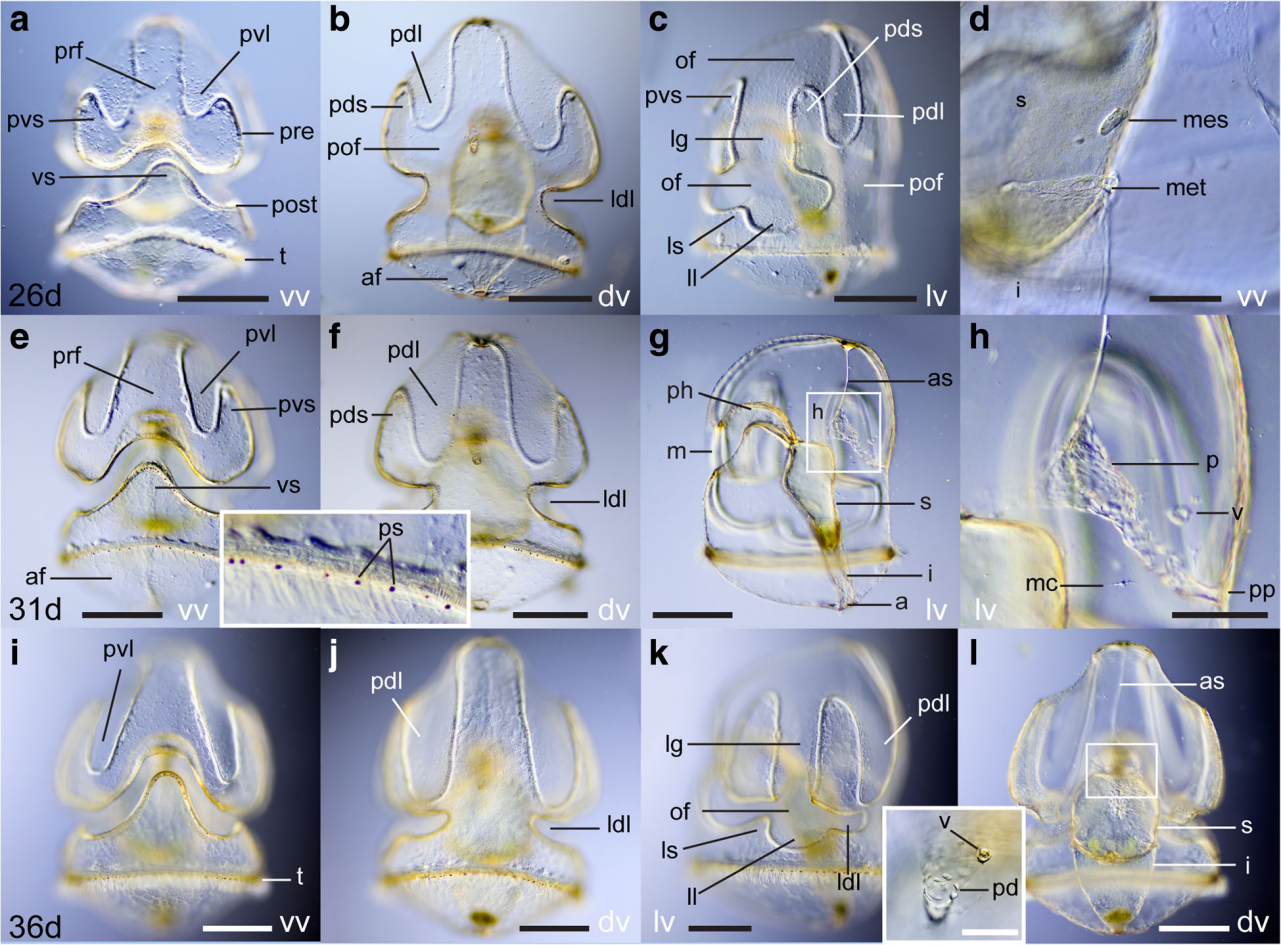
Morphology of the tornaria larva of *Schizocardium californicum* at 26, 31 and 36 dpf. Light DIC micrographs of live larvae. All panels are anterior to the top and lateral views are ventral to the left. Optical sections through the middle of the larva (**d, g**-**h, l)** or **s**urface views (**a**-**c**, **e**-**f**, **i**-**k**). **a**-**d** Tornaria larva at 26 dpf. **d** Close-up ventral view of the mesocoels and metacoels around the posterior stomach. **e**-**h** Tornaria larva at 31 dpf. Inset is a close-up of the telotroch showing pigment spots. **h** Close-up view of area boxed in panel g showing formation of the proboscis vesicle. **i**-**l** Tornaria larva at 36 dpf. Inset is a close-up view of the region highlighted in **l**, showing movement of the proboscis vesicle closer to the protocoel. as, apical strand; dv, dorsal view; i, intestine; ldl, lower dorsal lobe; ll, lateral lobe; ls, lateral saddle; lv, lateral view; m, mouth; mc, mesenchymal cell; of, oral field; pd, protocoel duct (pore canal); pdl, primary dorsal lobe; pds, primary dorsal saddle; ph, pharynx; pof, postoral field; post, postoral loop of the circumoral ciliary band; pp, protocoel pore (hydropore); pre, preoral loop of the circumoral ciliary band; ps, pigment spots; pvl, primary ventral lobe; pvs, primary ventral saddle; s, stomach; t, telotroch; v, proboscis vesicle; vs, ventral saddle; vv, ventral view. Scale bars: 400 μm (a-c, e-g, i-l), 100 μm (d, l inset), 200 μm (h)

**Fig. 7:**
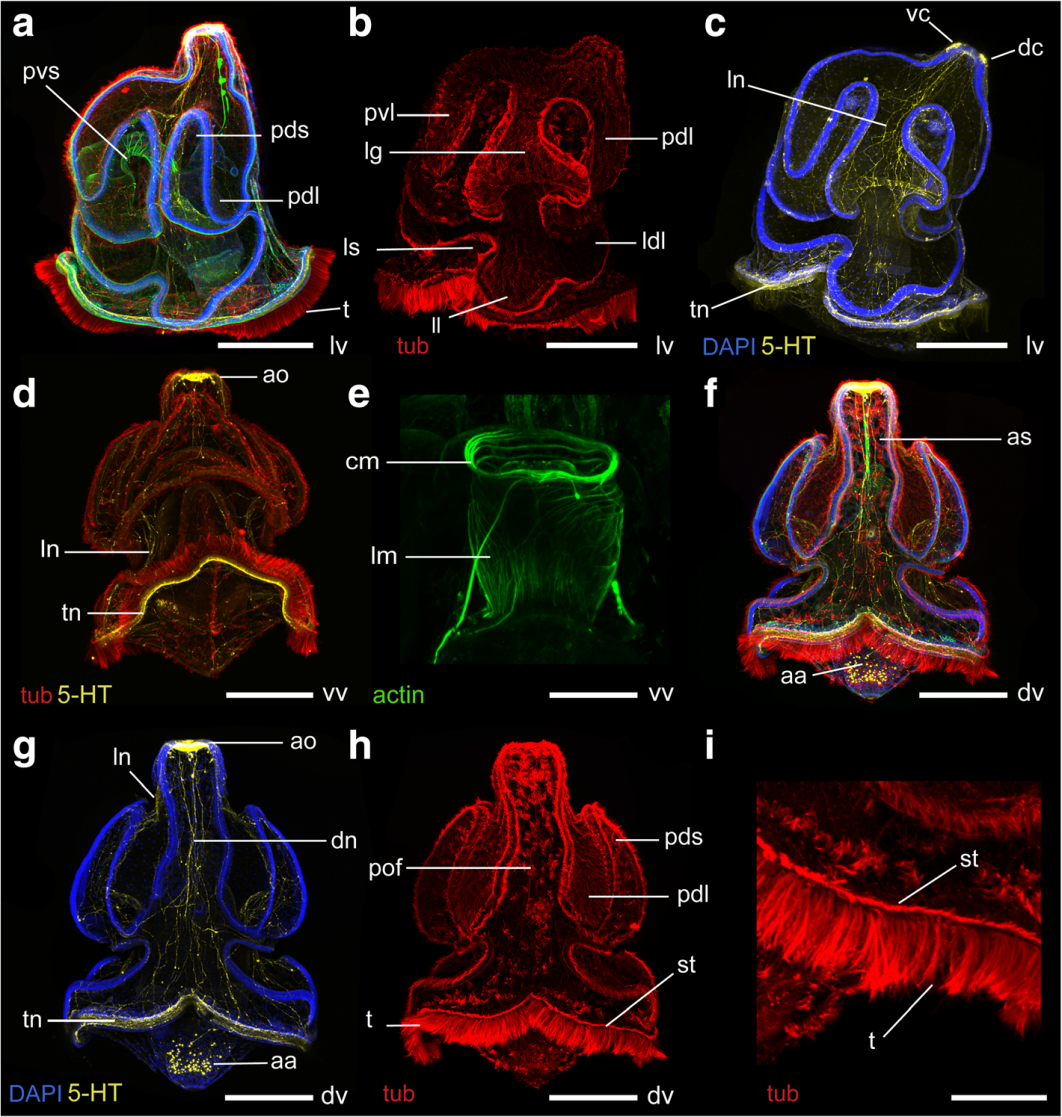
Morphology of the tornaria larva of *Schizocardium californicum* at 36 dpf. Maximum intensity projections of confocal stacks. Larvae are labeled with four markers: phalloidin (green), anti-acetylated tubulin (red) and anti-serotonin (yellow) antibodies, and DAPI (blue) unless otherwise indicated. All panels are anterior to the top and lateral views are ventral to the left. **a**-**c** lateral views. **b** Tubulin channel only, showing organization of the ciliary bands. **c** DAPI and serotonin channels only, showing the lateral nerve tract passing through the lateral groove and the telotroch nerve tract. **d** Ventral view, acetylated tubulin and serotonin channels only showing the nerve tract underlying the telotroch. **e** Close-up ventral view of the pharynx, phalloidin channel only, showing the circular and longitudinal muscles in the anterior and posterior pharynx, respectively. **f**-**i** Dorsal views. **g** DAPI and serotonin channels only, showing the dorsal nerve tract descending along the dorsal midline, and the telotroch nerve tract. **h** Acetylated tubulin channel only, showing organization of the ciliary bands. **i** Close-up view of the telotroch, showing the shorter cilia of the secondary telotroch. aa, autofluorescence caused by algal cells in digestive tract; ao, apical organ; as, apical strand; cm, circular pharyngeal muscles; dc, dorsal cluster of the apical organ; ldl, lower dorsal lobe; ll, lateral lobe; lm, longitudinal pharyngeal muscles; ln, lateral nerve tract; ls, lateral saddle; pdl, primary dorsal lobe; pds, primary dorsal saddle; pm, pharyngeal muscles; pof, postoral field; pvl, primary ventral lobe; pvs, primary ventral saddle; st, secondary telotroch; t, telotroch; tn, telotroch nerve tract. vc, ventral cluster of the apical organ. Scale bar: 500 μm (a-d, f-h), 200 μm (e, i)

Between 26 and 36 dpf (Fig. 6), the larva measures between 1.2 and 1.7 mm. Throughout this period, the length of the circumoral ciliary band continues to increase and its shape becomes more convoluted (Fig. 6a-c, e-f, i-k, Fig. 7). Folding of the preoral loop creates primary ventral saddles and primary ventral lobes (Fig. 6a, c, e, i, Fig. 7a-b). Dorsally, the left and right sides of the postoral loop move closer to each other around mid-length of the AP axis, creating the lower dorsal lobe, a groove that divides the dorsal ectoderm into clear preoral and postoral regions (Fig. 6b, f, j, Fig. 7b). Throughout this period, the lateral groove becomes narrower (Fig. 6c, k) and the postoral loop of the circumoral ciliary band develops additional folds laterally, the lateral lobes and lateral saddles (Fig. 6c, k, Fig. 7b). Pigmented spots begin to appear along the telotroch and circumoral ciliary band (Fig. 6e and inset).

At 26 dpf, the paired mesocoels and metacoels begin to develop (Fig. 6d). The metacoels develop as crescent-shaped masses of cells that originates at the boundary between intestine and stomach, and extend dorsally around the surface of the stomach. The smaller mesocoels appear shortly after and are closely connected to the stomach walls.

Around 30 dpf, the pulsatile vesicle of the pore canal-hydropore complex appears. It initially forms in the blastocoelar space between the protocoel and the dorsal ectoderm, on the right side (Fig. 6g-h), and slowly moves towards the protocoel and pore canal (Fig. 6l, inset).

At 36 dpf, the musculature is similar to the 22 dpf larva (Fig. 7e-f). A new band of cilia running immediately anterior to the telotroch develops, termed the secondary telotroch (Fig. 7h-i).

At 36 dpf, the serotonergic nervous system is essentially similar to the 22 dpf larva, with the addition of many neurites in the bundle that underlies the telotroch (Fig. 7c-d, g). Unlike the telotroch, there are no neurites specifically associated with the circumoral ciliary band. Instead, the neurites that descend laterally from the apical organ pass between the ventral and primary lobes along the lateral grooves (Fig. 7c, g). Dorsal projections from the apical organ run along the dorsal midline of the postoral field (Fig. 7g). Neurites are less abundant in the ectoderm of the primary lobes (Fig. 7g). Only a few neurites are seen posterior to the telotroch (Fig. 7c, d, g).

**Fig. 8:**
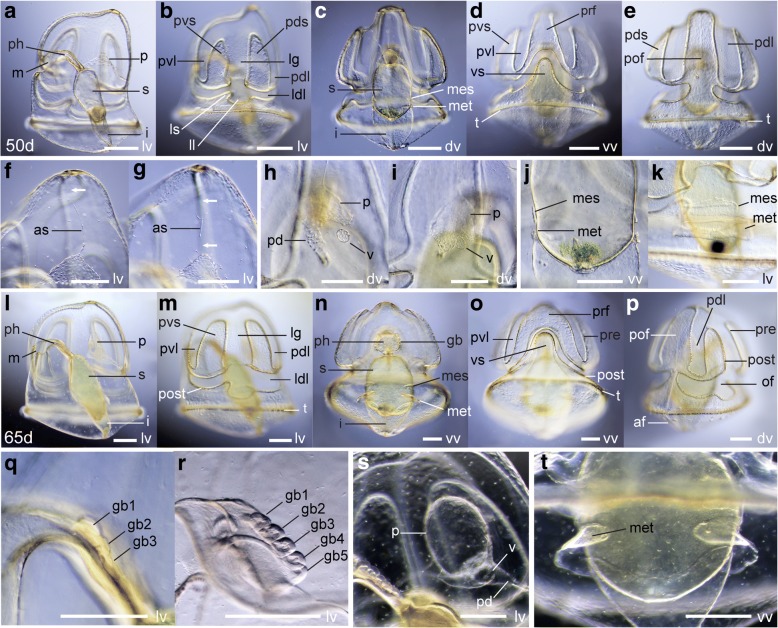
Morphology of the tornaria larva of *Schizocardium californicum* between 50 dpf and the end of the larval stage. Light DIC micrographs of live larvae. All panels are anterior to the top and lateral views are ventral to the left. **a**-**k** Tornaria larva at 50 dpf. **b**, **d**-**e** Surface views showing external morphology and organization of the ciliary bands. **a, c** Optical sections through the middle of the larva, showing internal anatomy. **f**-**g** Close-up view of the anterior end of the larva, showing regression of the apical strand. Arrows indicate where cells of the apical strand are no longer present. **h**-**i** Dorsal view of the protocoel region, showing the gradual movement of the proboscis vesicle towards the protocoel. **j**-**k** Growth and shape change of the posterior coeloms between 50 dpf (j) and 65 dpf (k). **l**-**p** Tornaria larva at 65 dpf. **l**-**n** Optical sections through the middle of the larva, showing internal anatomy. **m**, **o**-**p** Surface views showing external morphology and organization of the ciliary bands. **q**-**t** Morphological changes occurring between 65 dpf and metamorphosis. **q**-**r** Close-up view of the pharynx, showing development of gill bars. **s** Close-up view of the protocoel in its final form before metamorphosis. **t** Close-up view of the metacoel in its final form before metamorphosis. as, apical strand; gb, gill bar; i, intestine; ldl, lower dorsal lobe; lg, lateral groove; ll, lateral lobe; m, mouth; mes, mesocoel; lv, lateral view; met, metacoel; of, oral field; og, oral groove; p, protocoel; pd, protocoel duct (pore canal); pdl, primary dorsal lobe; pds, primary dorsal saddle; ph, pharynx; pof, postoral field; post, postoral loop of the circumoral ciliary band; pre, preoral loop of the circumoral ciliary band; prf, preoral field; pvl, primary ventral lobe; pvs, primary ventral saddle; s, stomach; t, telotroch; v, proboscis vesicle; vs, ventral saddle. Scale bars: 400 μm (a), 200 μm (f-k, q-t), 800 μm (l)

**Fig. 9:**
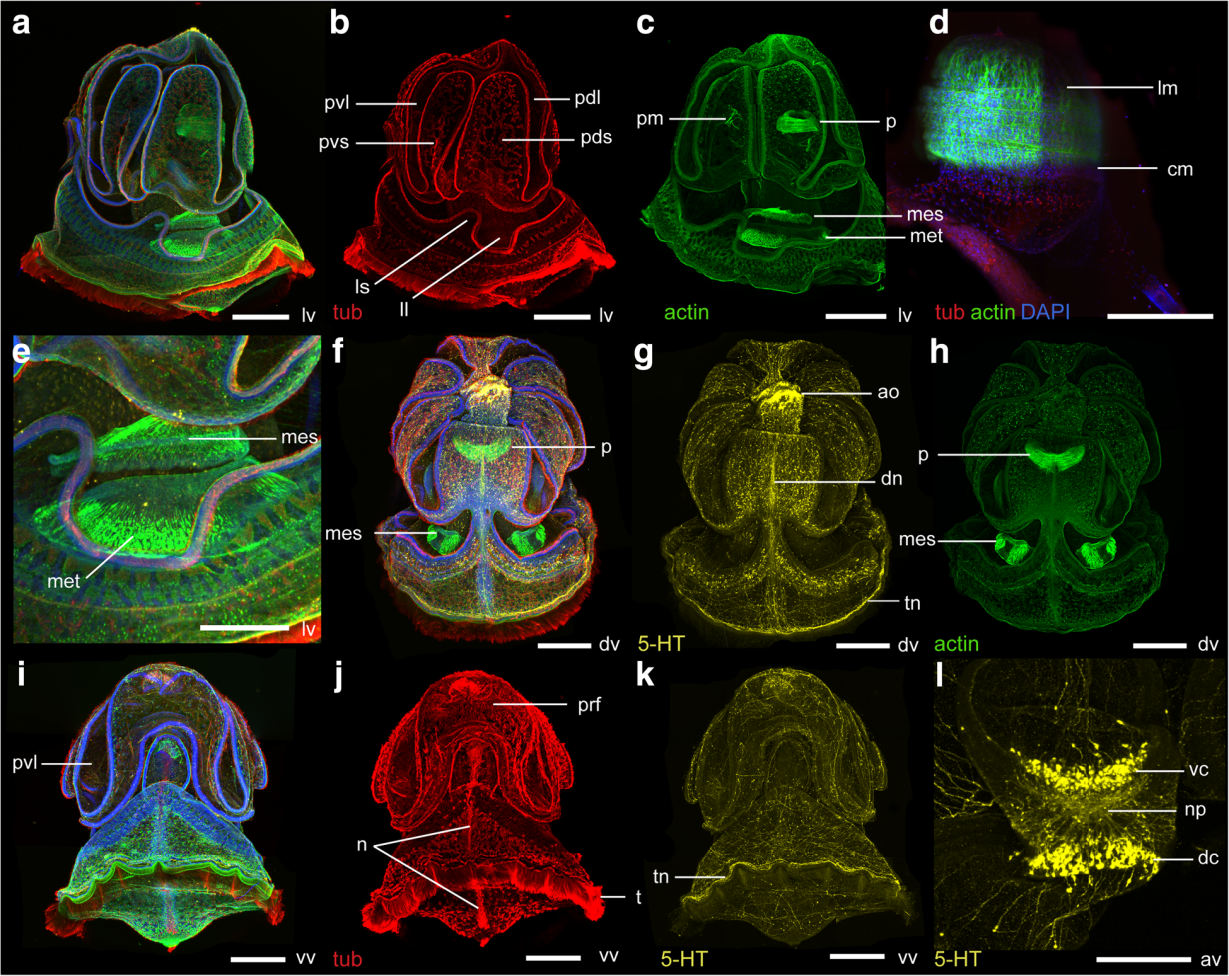
Morphology of the late tornaria larva prior to metamorphosis. Maximum intensity projections of confocal stacks. Larvae are labeled with four markers: phalloidin (green), anti-acetylated tubulin (red) and anti-serotonin (yellow) antibodies, and DAPI (blue) unless otherwise indicated. Anterior to the top and ventral to the left. **a**-**e** Lateral view. **b** Tubulin channel only, showing organization of ciliary bands. **c** Phalloidin channel only, showing highly muscularized coeloms and reduced pharyngeal musculature. Note that the apical strand is no longer present. **d** Close-up side view of the protocoel, showing circular and longitudinal muscle fibers. **e** Close-up side view of the left mesocoel and metacoel. **f**-**h** Dorsal view. **g** Serotonin channel only, showing dense epidermal neural net. **h** Phalloidin channel only, showing highly muscularized coeloms. **i**-**k** Ventral view. **j** Tubulin channel only, showing organization of ciliary bands. **k** Serotonin channel only, showing dense epidermal neural net. **l** Close-up anterior view of the apical organ, serotonin channel only. ao, apical organ; av, anterior view; cm, circular muscle fibers; dc, dorsal cluster of the apical organ; dn, dorsal nerve tract; dv, dorsal view; ll, lateral lobe; lm, longitudinal muscle fibers; ls, lateral saddle; mes, mesocoel; met, metacoel; n, neurotroch; np, neuropil; p, protocoel; pdl, primary dorsal lobe; pds, primary dorsal saddle; pl, primary lobe; pm, pharyngeal muscles; post, post-oral loop of the circumoral ciliary band; pre, pre-oral loop of the circumoral ciliary band; prf, pre-oral field; pvl, primary ventral lobe; pvs, primary ventral saddle; sv, side view; t, telotroch; tn, telotroch nerve tract; vc, ventral cluster of the apical organ. Scale bar: 600 μm (a-c, f-k), 300 μm (d-e, l)

Between 50 and 65 dpf, the larva reaches its final form (Figs. 8, 9). The primary dorsal and ventral lobes expand until the primary saddles almost close off (Fig. 8b, d-e, m, o-p, Fig. 9a-c, i-j). The left and right lower dorsal lobes extend until they almost meet on the dorsal side (Fig. 8p, 9b). The ventral saddle continues to expand anteriorly (Fig. 8d, o).

During this time period, the apical strand disappears (Fig. 8f-g, Fig. 9c). The pulsatile vesicle makes contact with the protocoel and pore canal on the right side (Fig. 8h-i). Interestingly, the protocoel duct bends towards the left before the pulsatile vesicle reaches it (Fig. 8h). Addition of the pulsatile vesicle to the pore canal-hydropore complex transforms the larval protonephridium into a muscle-driven metanephridial system [58]. Between 50 and 65 dpf, the mesocoels and metacoels continue to increase in size (Fig. 8j-k).

After 65 dpf, the larva undergoes the last series of changes before metamorphosis. The larva keeps growing until it reaches about 3 mm in length. The gill slits appear as paired perforations of the pharyngeal walls, surrounded by oval-shaped endodermal gill bars. Gill slits appear sequentially, following an anterior to posterior progression (Fig. 8q-r). Five or six pairs are present in the tornaria larva in its final form. In the tornaria larva, only the endodermal components of the gill slits are present, as the ectodermal gill pores that connect the pharynx lumen to the external environment only appear later during metamorphosis (see below). During this period, all coeloms grow in size (Fig. 8s-t), and become muscularized (Fig. 9a, c-f). Finally, the serotonergic nervous system undergoes dramatic changes. Many serotonergic cell bodies and neurites appear throughout the postoral field and primary lobes, including under the circumoral ciliary band (Fig. 9g, k). A neurite bundle runs along the dorsal midline (Fig. 9g). At this stage, the apical organ remains similar to earlier stages, but is now composed of dozens of cell bodies, joined by a dense neuropil (Fig. 9l). Unlike most ptychoderid species, the tornaria of *S. californicum* never develops secondary lobes and saddles.

After the larva undergoes these last changes, it is ready to metamorphose. Under laboratory conditions, metamorphosis may occur anytime between 2 and 8 months (possibly more), depending on feeding rate and population density. Under high abundance of food and low population density, larvae start metamorphosing spontaneously after 2 months. Onset of metamorphosis is highly variable between larvae raised under identical conditions, as individuals raised in the same container often initiate metamorphosis at intervals up to several weeks.

When maintained at high densities and low food regimes, larvae were kept up to 8 months before metamorphosis was observed, with no visible morphological changes during this period.

### Metamorphosis

**Fig. 10:**
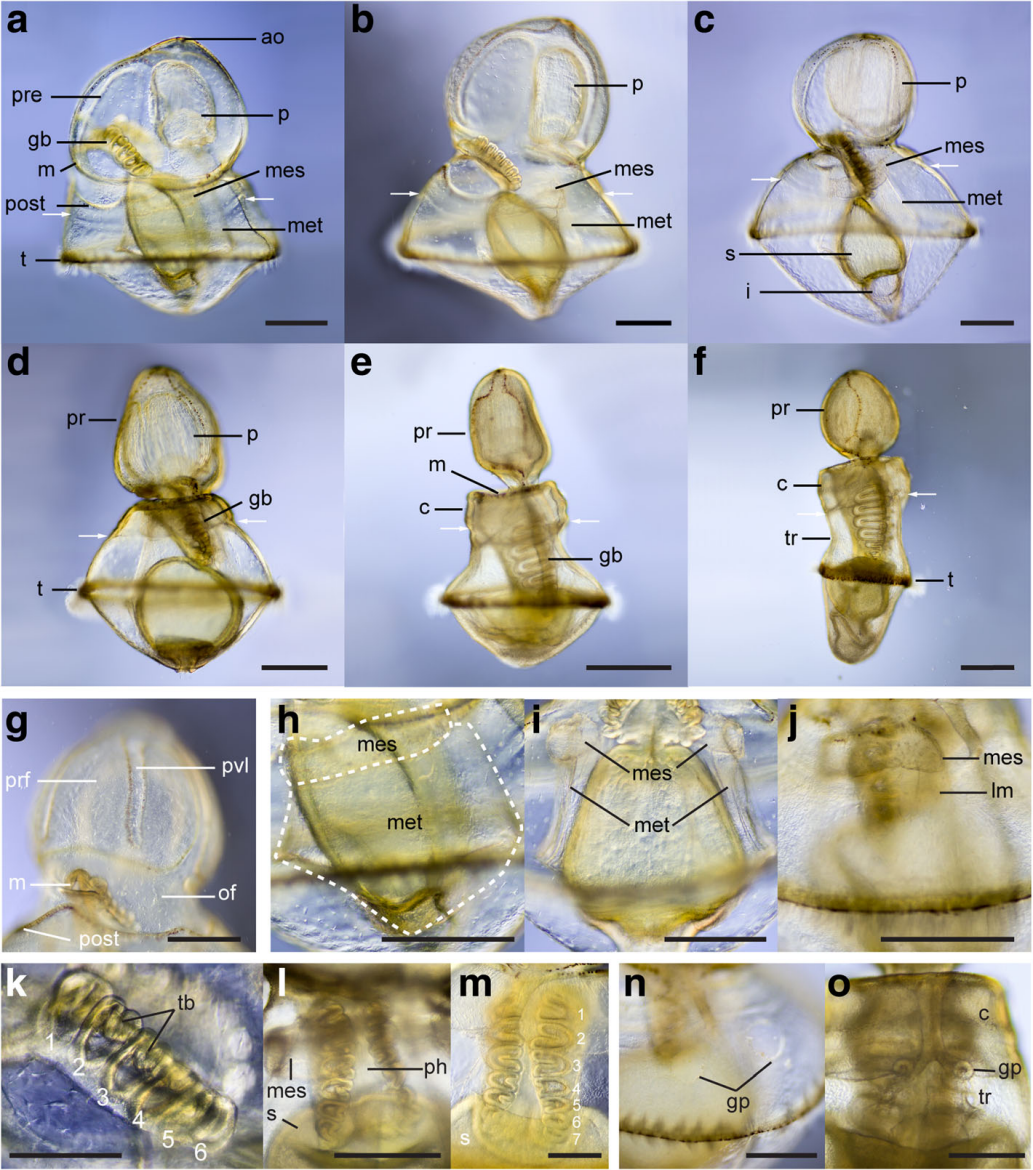
Metamorphosis in *Schizocardium californicum*. Light DIC micrographs of live individuals. Anterior to the top and ventral to the left. **a** Larva at onset of metamorphosis, showing enlarged coelomic cavities**. b**-**c** Larvae between 3 h and 5 h after onset of metamorphosis, showing thickening of the ectoderm, posterior retraction of the pharynx, and further enlargement of coelomic cavities. **d** Larva around 10–12 h after onset of metamorphosis, showing almost fully formed proboscis, thickening collar, and folding of the gut. **e** Larva around 24 h after onset of metamorphosis, showing fully formed proboscis and collar, further folding of the gut, enlargement of pharynx and gill slits, and formation of the trunk. **f** Post-metamorphic juvenile, 48 h after onset of metamorphosis, showing fully formed proboscis and collar, and elongating trunk. **g** Close-up ventral view of the forming proboscis. Same individual as **c**. The anterior ciliary grooves are disappearing as the loops of the circumoral ciliary band begin to fuse. **h**-**j** Changes in coelomic organization during metamorphosis. **h** Onset of metamorphosis. Same individual as **a**. **i** 5 h after onset of metamorphosis. **j** 12 h after onset of metamorphosis. The metacoels are forming the longitudinal muscles of the trunk. **k**-**m** Changes in pharyngeal morphology during metamorphosis. **k** Same individual as **a**, showing 6 gill slits at onset of metamorphosis, and the presence of tongue bars. **l** pharynx at 12 h after onset of metamorphosis, showing relative position with the mesocoels. **m** Pharynx of larva around 24 h after onset of metamorphosis, showing addition of a seventh pair of gill slits. **n**-**o** Ectodermal gill pore formation. **n** Same individual as **c**. **o** Same individual as **e**. ao, apical organ; c, collar; gb, gill bars; gp, gill pores; i, intestine; lm, longitudinal trunk muscle; m, mouth; mes, mesocoel; met, metacoel; of, oral field; p, protocoel; ph, pharynx; post, postoral loop of the circumoral ciliary band; pr, proboscis; pre, preoral loop of the circumoral ciliary band; prf, preoral field; pvl, primary ventral lobe; s, stomach; t, telotroch; tb, tongue bar; tr, trunk. White arrows mark the posterior boundary of forming collar. Scale bars: 500 μm (a-j, l-n), 250 μm (k, o)

**Fig. 11:**
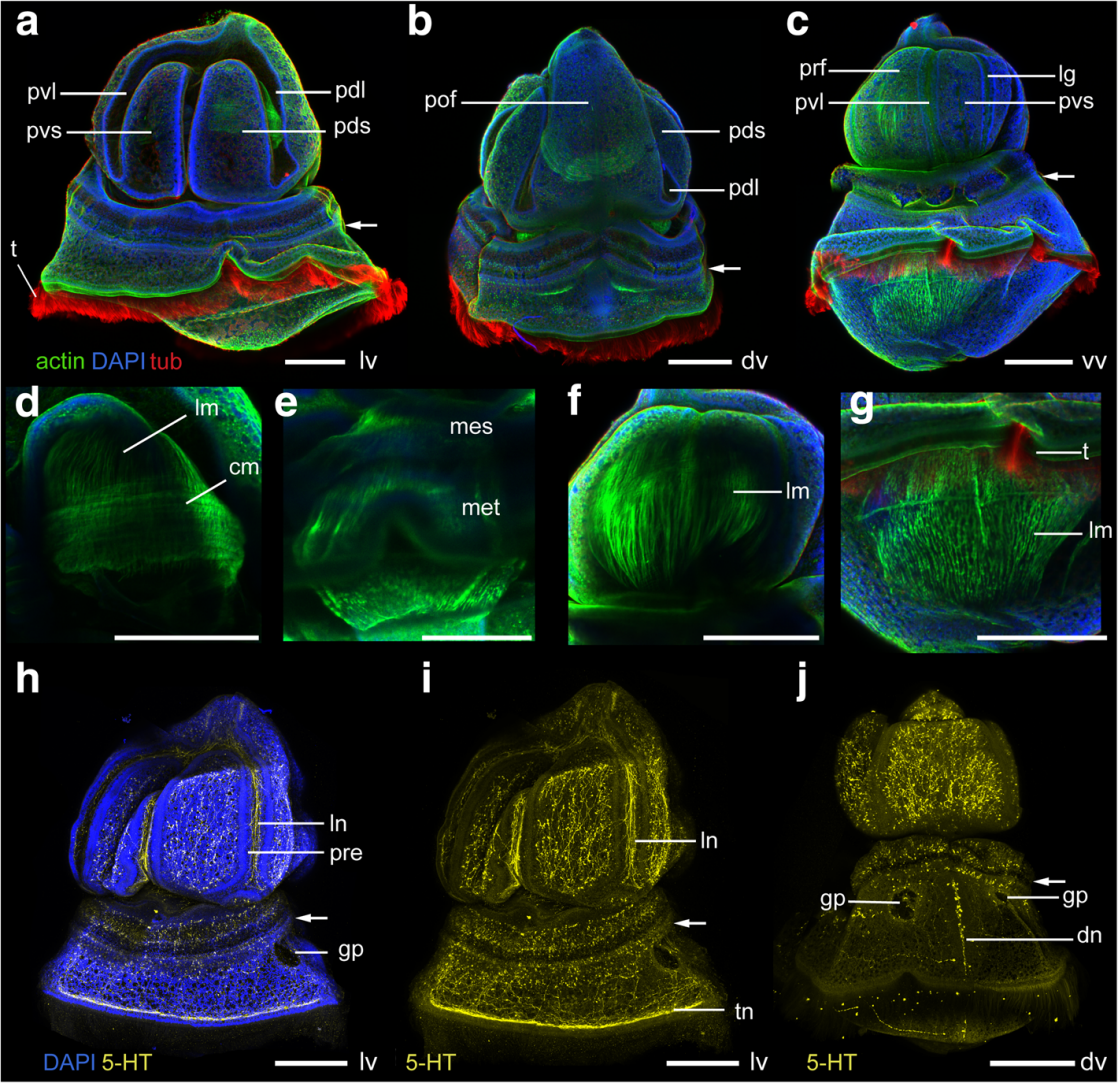
Early metamorphosis in *Schizocardium californicum*. Maximum intensity projections of confocal stacks. **a**-**g** Larvae labeled with phalloidin (green), anti-acetylated tubulin antibody (red) and DAPI (blue). **h**-**j** Larvae labeled with anti-serotonin (yellow) antibodies, and DAPI (blue). All panel are anterior to the top and lateral views are ventral to the left. **a** Onset of metamorphosis, same stage as Fig. 10a. **b** Larva during early metamorphosis, same stage as Fig. 10b. The primary lobes are disappearing as the loops of the circumoral ciliary band begin to move toward each other. **c** Larva during mid-metamorphosis, similar stage as Fig. 10c. **d**-**g** Close-up views of coeloms and associated musculature, same individuals as panel a (**f**-**g**) and panel c (**d**-**e**). **h**-**i** Serotonergic nervous system during early metamorphosis (similar stage as panel b), showing neurites and cell bodies scattered throughout the future proboscis, collar and anterior trunk down to the telotroch. Neurite bundles are still present in the lobes and lateral grooves of the forming proboscis. **j** Serotonergic nervous system during early metamorphosis (similar stage as panel c). Neurite bundles are no longer present in the lobes and lateral grooves. Neurites of the anterior trunk are less abundant. A dorsal nerve tract runs along the trunk midline. cm, circular muscle fibers; dn, dorsal nerve tract; dv, dorsal view; gp, gill pore; lm, longitudinal muscle fibers; ln, lateral nerve tract; lv, lateral view; mes, mesocoel; met, metacoel; pdl, primary dorsal lobe; pds, primary dorsal saddle; pof, postoral field; prf, preoral field; pvl, primary ventral lobe; pvs, primary ventral saddle; t, telotroch; tn, telotroch nerve tract; vv, ventral view. Scale bars: 500 μm

The first sign of metamorphosis is an increase in the size of the coelomic cavities (Fig. 10a, h, Fig. 11a, d-e) and a thickening of the ectoderm, which makes the larva more opaque.

In the following three to five hours (Fig. 10a-c), the protocoel extends anteriorly and expands to fill most of the preoral cavity (Fig. 10a-b, Fig. 11d, f). Simultaneously, the mesocoels and metacoels expand both along the dorsoventral and anteroposterior dimensions (Fig. 10a-c, h-i, Fig. 11e, g). Before metamorphosis, both pairs of posterior coeloms are attached to the posterior half of the stomach, at the same level of the AP axis as the telotroch (Fig. 8n, t). As they expand at the beginning of metamorphosis, their position relative to the endoderm and ectoderm shifts. The mesocoels move anteriorly, until they end up being located under the postoral loop of the circumoral ciliary band, and about a third of the way from the anterior end of the stomach (Fig. 10a, h). The metacoels surround most of the stomach between the mesocoels and the posterior endoderm. Shortly after, the endoderm begins to move posteriorly (Fig. 10b). As a result, the mesocoels become located around the boundary between pharynx and stomach (Fig. 10b, i). Above the mesocoels, the ectoderm of the future collar, marked anteriorly by the postoral loop of the circumoral ciliary band, begins to thicken (Fig. 10b). The future collar-trunk boundary becomes visible and is marked by a difference in thickness of the ectoderm and a slight constriction (Fig. 10a-f, arrows). Until the end of metamorphosis, the metacoels extend from this region down to the posterior stomach (Fig. 11e).

As the pharynx retracts from the preoral body cavity, the protocoel expands rapidly (Fig. 10c). Meanwhile, the ectoderm of the preoral lobe (the future proboscis) thickens and condenses over the expanding underlying anterior mesoderm. Most of this decrease in size is at the expense of the oral field, which gradually disappears as the loops of the ciliary band come closer together (Fig. 10g, Fig. 11b-1c). At this stage, a pair of large openings forms in the dorsal ectoderm (Fig. 10n, Fig. 11h-j). These are the future ectodermal gill pores that will later connect the pharyngeal slits to the external environment.

During the following six hours, the pharynx continues to retract until it is located entirely posterior to the mouth (Fig. 10d). The space in the postoral body cavity is insufficient for both pharynx and stomach, and the pharynx begins to fold over the dorsal stomach. During this period, the ectoderm of the proboscis continues to thicken, and comes in close proximity to the enlarged protocoel (Fig. 10d, Fig. 11c, f). All of the larval ectoderm corresponding to extensions of the oral field in the future proboscis (dorsal lobe, ventral lobe and lateral groove) disappear completely, as the loops of the circumoral ciliary bands eventually fuse and close off these ectodermal domains. The pigmented spots of the ciliary band remain, and mark the position where the loops fuse. These pigmented spots remain visible for days. Similar to the proboscis ectoderm, the ectoderm of the future collar thickens and becomes clearly recognizable as a distinct body region (Fig. 10d). At that stage, the protocoel and metacoels begin to form the musculature of the juvenile (Fig. 11f-g), and the circular muscles of the pharynx disappear.

**Fig. 12:**
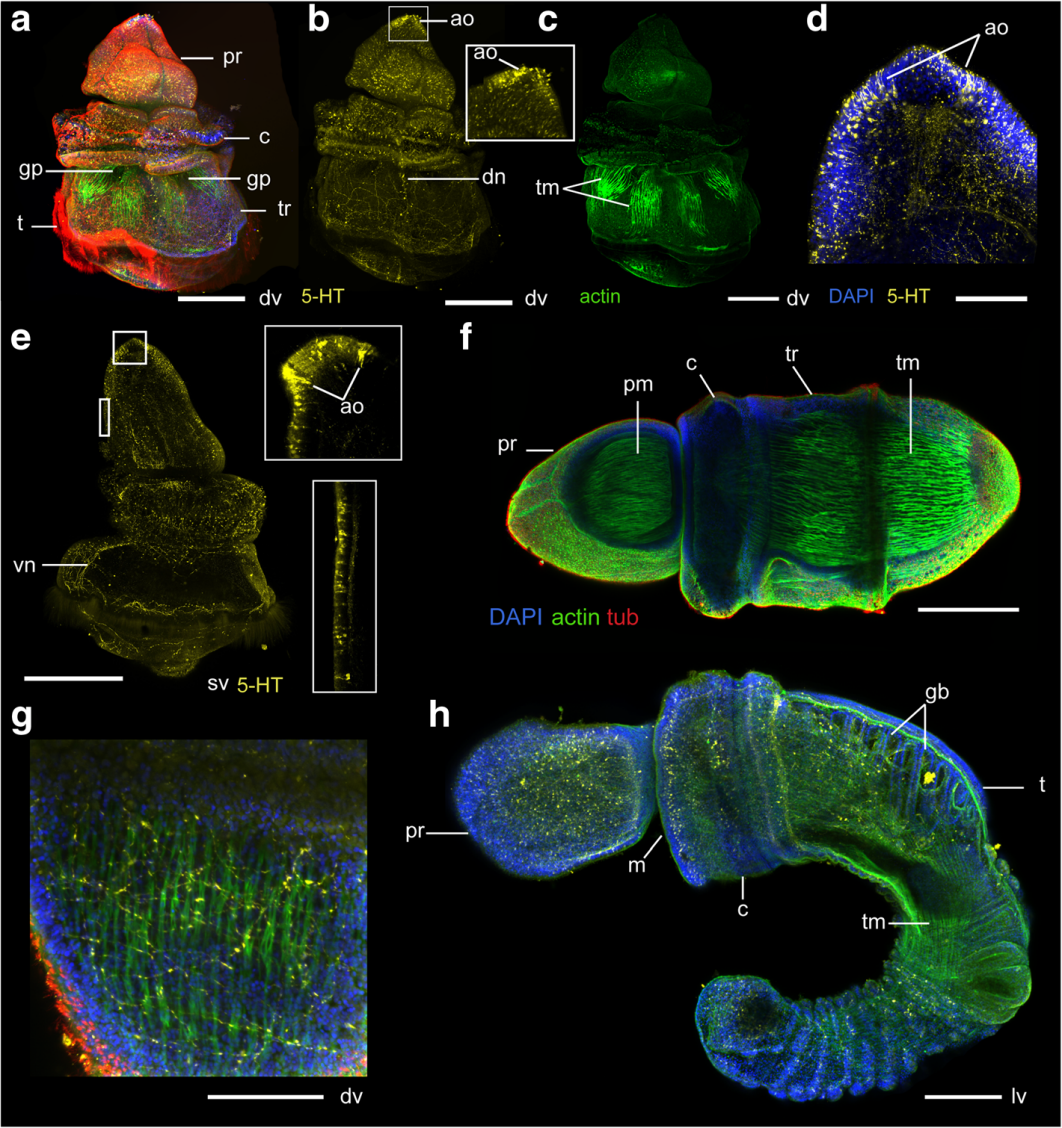
Late metamorphosis and early juvenile development in *Schizocardium californicum*. Larvae are labeled with four markers: phalloidin (green), anti-acetylated tubulin (red) and anti-serotonin (yellow) antibodies, and DAPI (blue) unless otherwise indicated. **a**-**e** 24 h after onset of metamorphosis. Anterior to the top. **b** Serotonin labeling only. Inset shows a close-up on the larval apical organ, still visible. **c** Phalloidin labeling only, showing longitudinal muscles in the trunk. **d** Close-up of the anterior proboscis, serotonin and DAPI labeling only. Neurons of the larval apical organ are still visible, but become less distinct from the broadly distributed neurons of the juvenile proboscis as time progresses. **e** Serotonin labeling only. Insets show optical slices through the anterior tip and ventral side of the proboscis epidermis. **f** Early juvenile, 48 h after onset of metamorphosis. Surface optical slices are excluded from the stack, showing muscles inside the proboscis and trunk. **g** Close-up view of the trunk epidermis, showing basiepidermal serotonergic nerve net and muscle fibers underlying the ciliated epithelium. **h** Juvenile, 15 days post-metamorphosis. ao, apical organ; c, collar; dn, dorsal neurite bundle; dv, dorsal view; gb, gill bars; gp, gill pore; lv, lateral view; m, mouth; pm, proboscis muscles; pr, proboscis; t, telotroch; tm, longitudinal trunk muscles; tr, trunk; vn, ventral neurite bundle. Scale bars: 500 μm (a-c, e-f, h), 100 μm (d), 200 μm (g)

Over the next twelve hours (Figs. 10e and 12a-e), the remaining blastocoelar space in the proboscis disappears. Similarly, the ectoderm of the future collar thickens and becomes tightly apposed to the mesocoels. The same process begins in the future trunk, and this transformation follows an anterior to posterior temporal progression. In the anterior trunk, the first two gill pores decrease in size and become surrounded by a circular thickening of the ectoderm. (Fig. 10o). Meanwhile, the pharynx grows both posteriorly and ventrally, and the size of the gill slits increases dramatically. The pharynx grows over the dorsal stomach, which folds into a S-shape (Fig. 10e). At that time, the longitudinal muscles of the trunk are well developed (Figs 10j and 12c). The cilia of the telotroch keep beating, and the larva keeps swimming until the end of metamorphosis.

Over the next 24 h (Figs. 10f, and 12f), the trunk continues to collapse over the metacoels and gradually elongates, both anterior and posterior to the telotroch. Gill slits continue to form (Fig. 10k-m). At this stage, the juvenile has a clear proboscis, collar and trunk, and is able to burrow and start its benthic life.

Throughout metamorphosis, the serotonergic nervous system undergoes extensive reorganization. Cell bodies of the apical organ remain visible until late metamorphosis (Fig. 12b, d-e), but are gradually lost in the apical organ. During this time period, many additional serotonergic neurons appear throughout the epidermis of the proboscis, and the boundaries between the apical organ and the general nervous system of the proboscis become less well defined (Figs. 11j and 12b, d-e). These neurons resemble the bipolar neurons described in both *Saccoglossus kowalevskii* and *B. simodensis* [30]. Additionally, the neurite bundles that are present along the ciliary grooves at the beginning of metamorphosis disappear as the ciliary bands fuse (Fig. 11i-j). Additional serotonergic neurons appear in the collar, and the cell bodies and nerve plexus of both the forming proboscis and collar likely contribute to the nervous system of the juvenile (Fig. 12g, h). In the forming trunk, many serotonergic neurites disappear but the cell bodies and axon tracts on the dorsal and ventral midlines remain (Fig. 11i, j, Fig. 12b, e).

## Discussion

### *Schizocardium californicum* is a suitable species for developmental biology

Many species of enteropneusts present practical challenges for developmental studies, due to short reproductive seasons, remote collecting areas, and difficulties in inducing spawning. In addition, indirect developing species require a long period of larval rearing, and it is often difficult to rear the larvae through to metamorphosis. Many of these issues have been solved only recently in *P. flava*, a ptychoderid worm from the Indo-Pacific [22]. Here, we show that *Schizocardium californicum* is an easily accessible, temperate species suitable for developmental biology. While its natural reproductive cycle remains to be characterized in detail, we report that this species can be spawned under laboratory conditions almost year-round. In *P. flava*, the reproductive season is limited to July–December [22], and in *Saccoglossus kowalevskii* to September and May. Spawning in *Schizocardium californicum* can be triggered using a simple heat shock treatment. Unlike *Saccoglossus kowalevskii*, we cannot use the timing of heat shock to reliably control spawning time [13], but spawning induction is more straight-forward than in *P. flava*, where treatment with sperm water and a pre-cooling period are required [22]. Another convenient feature of *Schizocardium californicum* is that active sperm can be directly dissected out of the testes, without the need to induce natural sperm release as is necessary in *P. flava*. The extremely large numbers of eggs produced in *Schizocardium californicum* is convenient for easily generating large larval cultures, providing large amounts of material necessary for experimental work. Under our culture conditions, metamorphosis occurs spontaneously when the larva reaches its maximal size, starting around two months after fertilization. Unlike *P. flava*, exposure to substrate from the collecting site is not necessary to induce metamorphosis.

### Comparison with other indirect developing enteropneusts

Below, we compare development in *Schizocardium californicum* with published data from other indirect developing enteropneusts. Studies between the late 1800s and the 1950s have described or reported indirect development in six described ptychoderid species, in a number of ptychoderid tornaria larvae of uncertain taxonomy collected from the plankton, and in two spengelids (reviewed in [14]). More recent descriptions of indirect development were made in *P. flava*. These descriptions include light micrographs of early development [26] or of the full life cycle [22], and investigations of the neuroanatomy [24]. Additionally, indirect development from embryogenesis to metamorphosis was described using light microscopy in two additional ptychoderids, *Balanoglossus simodensis* [27, 28] and *Balanoglossus misakiensis* [29, 30], and in an additional spengelid, *Glandiceps hacksi* [32]. The nervous system of pre- and post-metamorphic stages of *B. misakiensis* was investigated using immunolabeling and confocal microscopy [30].

### Embryonic and larval stages

Egg size in *Schizocardium californicum* (110 μm) is typical of indirect developing enteropneusts, where egg diameter ranges from 60 to 160 μm [14]. Early cleavage is similar to other enteropneusts with the exception of the fourth cleavage, which is along the A/V axis in other species [21] while it is equatorial in *Schizocardium californicum*, resulting in a 16-cell stage embryo where animal blastomeres are arranged in two tiers of four cells on top of each other. The 16-cell stage is not described in the descriptions of *G. hacksi* [32], and early cleavage has not been described in the previous account of larval development in *Glandiceps* [31]. Whether it is common to other spengelids is therefore unknown.

Formation of a coeloblastula and gastrulation occur similarly in *Schizocardium californicum* as in other enteropneust species. In *Schizocardium californicum* and *G. hacksi* the protocoel pore and pore canal form shortly after gastrulation and before the protocoel itself, while in ptychoderids the protocoel pinches off from the archenteron before the pore canal and protocoel pore form [26]. Our observations therefore confirm that the “precocious hydropore formation” described in *G. hacksi* [32] may be a common feature of spengelids. In *Schizocardium californicum*, hatching occurs at 26 hpf, shortly after gastrulation and blastopore closure, but before formation of the pore canal. In *G. hacksi*, hatching occurs slightly later, after formation of the protocoel pore, but prior to formation of the protocoel [32]. In ptychoderids, hatching occurs after the protocoel, pore canal and protocoel pore are already apparent [26]. In all cases, hatching occurs before mouth formation.

### Ciliary bands

The changes in shape of the circumoral ciliary band are the main character used to distinguish the developmental stages of tornaria larvae. Early development of the ciliary bands in *Schizocardium californicum* is similar to *P. flava* [24]. Initially, the circumoral ciliary band is composed of two simple longitudinal loops and the telotroch is absent (Fig. 3) (Müller stage). Later, the neurotroch and telotroch develop (Fig. 4) (Heider stage), and are both made of multiciliated cells bearing long cilia. As the circumoral band increases in length, ventral and dorsal lobes divide the preoral and postoral fields respectively, resulting in the formation of primary saddles. Additionally, the lower dorsal lobe extends dorsally and divides the larva into a clear pre- and postoral regions. This process marks the transition to the Metschnikoff stage (Figs. 5-7).

In most species, further folding of the ciliary band results in the formation of secondary lobes and saddles, marking the transition to the Krohn stage. In *Schizocardium californicum*, these additional folds do not occur and the shape of the ciliary band remains relatively simple until metamorphosis (Figs. 8-9). In *P. flava*, secondary lobes and saddles are highly developed and the circumoral ciliary band becomes so convoluted that it forms ciliary lappets called tentacles. In the spengelid *G. hacksi* and the ptychoderids *B. simodensis* and *B. misakiensis* the situation is intermediate, with recognizable ventrolateral and dorsolateral grooves that do not reach the level of complexity seen in *Ptychodera*. Another variable trait among tornaria larvae is the presence of a secondary telotroch, which has been reported in three species of *Balanoglossus* [27, 29, 59]. This ciliary band is present in *S. californicum* but absent in *P. flava* and *G. hacksi*. The simple organization of the ciliary band in *Schizocardium californicum* in its final form is similar to a *P. flava* at the late Metschnikoff stage (Fig. 9a, b in [24]).

### Structures of the apical plate

The apical tuft of cilia appears at the same stage in *Schizocardium californicum* and *P. flava*, about two days after fertilization, around the time of mouth formation. Surprisingly, *G. hacksi* does not possess an apical tuft [32]. Our observations suggest that this is not a shared trait of spengelids. However, the apical tuft of *Schizocardium californicum* disappears between 18 and 22 dpf, while in *B. misakiensis* it is present until metamorphosis [30]. Unlike these indirect developing species, direct developing enteropneusts such as *Saccoglossus kowalevskii* retain their apical tuft long after the proboscis is fully formed (for example, see Fig. 4a in [60]) presumably reflecting the need for this sensory structure at the time of hatching. Interestingly, in *Saccoglossus kowalevskii* the apical tuft of cilia is not associated with an apical cluster of serotonergic neurons [30].

The pigmented eyespots in the apical plate of *Schizocardium californicum* develop at the same time as other described species. Ultrastructural studies in *Glossobalanus marginatus* have demonstrated that they are part of a photosensory system that includes rhabdomeric photoreceptor cells, and allows the larva to sense the direction of light [61]. As was the case in other studies [62], we observed phototactic behavior in early tornaria larvae, and negative phototaxis in late larval life prior to metamorphosis.

### Muscles

Development of the apical strand occurs similarly to *G. hacksi*, as the telotroch begins to form at around 5 dpf. In contrast the *P. flava* apical strand develops much earlier, shortly after protocoel formation [26]. We observed that contraction of the apical strand results in an inward retraction of the apical plate. This behavior has been observed in other species [25] but its function is unknown. The pharyngeal circular and longitudinal muscles make up the other main muscle group in the tornaria of *Schizocardium californicum*. Similar muscles function in rejecting food particles from the pharynx in *B. biminiensis* [63] but not in *P. flava* [25].

### Coeloms

In all tornaria larvae, the protocoel forms during early development, while the mesocoels and metacoels develop later, marking the transition to the Krohn stage. In *Schizocardium californicum*, mesocoels and metacoels develop identically to *G. hacksi* [32], where the metacoels appear as crescent-shaped groups of cells at the boundary of intestine and stomach, and mesocoels appear as flat patches on the external wall of the stomach. The coelomic cavities of these two species also have an identical shape at the end of larval development. This mode of coelom development differs from *Balanoglossus clavigerus*, *B. misakiensis* and surprisingly to another spengelid, *Glandiceps sp.* [31] where posterior coeloms first appear as single paired evaginations of the archenteron which later divide to give rise to distinct paired mesocoels and metacoels [14]. In addition to these differences, the relative contribution of mesenchymal cells and evagination from the endoderm seems to be variable between species [14], and suggests a high level of variation in mechanisms of coelomogenesis in indirect developing hemichordates. It is important to note that in *Schizocardium californicum*, the development of posterior coeloms does not coincide with the development of secondary lobes and saddles in the ciliary band (Table 1). Therefore, the tornaria of *Schizocardium californicum* in its final form possesses characters from both the Metschnikoff stage (ciliary band) and Krohn stage (posterior coeloms) of *P. flava*. This illustrates that differences in the relative timing of certain developmental events make the larval staging scheme established in early studies [55, 56] (and widely used thereafter) difficult to apply across all species.

### Nervous system

Elements of nervous system development have been described in several ptychoderids. In *P. flava*, the most complete developmental series was described by Nielsen and Hay-Schmidt [24] using immunolabeling against serotonin. This study describes early development until 6 days after hatching, as well as late and metamorphosing larvae from the plankton. The nervous system of *P. flava* was also described using an antibody against 1E11 (an echinoderm neural marker) in 7-day larvae [23]. Observations were made on larval stages collected from the plankton in *Balanoglossus proterogonius* using cholinesterase activity [64], and antibodies against FRMFamide and serotonin [65]. Synaptotagmin immunoreactivity was described throughout larval development and metamorphosis in *B. simodensis*, but its serotonergic nervous system was only described at a few isolated stages [28]. Finally, the serotonergic nervous system was described in Heider and Metschnikoff stage larvae [66] and during metamorphosis [30] in *B. misakiensis*. Our basic description of the serotonergic nervous system in *Schizocardium californicum* provides the first data on nervous system development in spengelids.

Our observations on early development of the serotonergic nervous system show both similarities and differences with available data at similar early stages in *P. flava* [24] and *B. simodensis* [28]. In both *P. flava* and *B. simodensis*, serotonergic neurons appear in the apical plate earlier than in *Schizocardium californicum*, at late gastrula stage. In both *P. flava* and *Schizocardium californicum*, additional serotonergic neurons appear progressively throughout larval development. In *P. flava*, serotonergic cell bodies develop both within the circumoral ciliary band and in the apical organ, while in *Schizocardium californicum*, serotonergic neurons are restricted to the apical organ until the larva reaches its final form.

As in all previously described tornaria larvae, the serotonergic neurons in the apical organ of *Schizocardium californicum* are organized in two distinct clusters located on the ventral and dorsal sides of the apical plate. These neurons send neurites into the neuropil that underlies the apical plate. Unlike *P. flava* [24], neurites that project posteriorly do not specifically innervate the circumoral ciliary band. Towards the end of metamorphosis, the serotonergic neurons are lost in the most apical region of the ectoderm as the number of cell bodies in the general proboscis ectoderm proliferates. This contrasts with juveniles of *B. misakiensis*, but is similar to *Saccoglossus kowalevskii.*

The presence of a serotonergic neurite bundle under the telotroch was described in *P. flava* during metamorphosis [24], and in Metschnikoff stage tornaria and metamorphosing larvae in *B. misakiensis* [30, 66]. In *Schizocardium californicum* its development coincides temporally with the appearance of the telotroch, suggesting that this neural structure may play a role in the control of swimming behavior. However, in *B. misakiensis* available data suggest that a well-developed telotroch may be present before the underlying neurite bundle becomes visible (Fig. 2.10a in [66]). At the end of larval development in *Schizocardium californicum*, the serotonergic nervous system includes cell bodies and neurites scattered throughout the epidermis. The neuroanatomy of *Schizocardium californicum* at the end of the larval stage resembles that of *B. misakiensis* at the Spengel stage (closer to metamorphosis) where an extensive basiepidermal serotonergic nerve plexus and scattered serotonergic cell bodies are present throughout most of the larval epidermis [30].

### Metamorphosis and early juvenile development

Recent descriptions of metamorphosis are available in *P. flava* [22], *B. misakiensis* [29, 66], and *B. simodensis* [27]. Traditional hemichordate literature describes indirect development in enteropneusts as a process of progressive development reaching a high point (the Krohn stage) followed by a period of regressive development (the Spengel and Agassiz stages) where some larval structures are lost, concluding in a sudden switch (metamorphosis stage) to the adult form and lifestyle (summarized in [14] and in Table 1).

The first step of regressive development involves the loss of secondary lobes and saddles and tentacles (when present), as well as a reduction in size and a thickening of the epidermis that makes the larva opaque. These traits define the Spengel stage, which can last for months in *P. flava* [22]. Unlike ptychoderids, the tornaria larva of *Schizocardium californicum* lacks secondary lobes and saddles. Additionally, in *S. californicum* a decrease in size and thickening of the epidermis is invariably followed by a continuous and rapid progression through all the steps of metamorphosis. As a result, there is no clearly defined, discrete Spengel stage. As in *Schizocardium californicum*, *B. misakiensis* lacks the secondary lobes and saddles characteristic of the Krohn stage. This led the authors to suggest that this species skips the Krohn stage and proceeds directly to the Spengel stage [29]. These observations illustrate that not all species fit the established staging scheme.

The traditional staging scheme defines the mid-point of the transition between larval and adult form (when the larva has a proboscis but is still swimming) as a discrete larval stage, called the Agasssiz stage, and narrowly defines metamorphosis as the final step of this transition, when the juvenile worm settles on the benthos. This definition excludes most of the crucial morphological changes from the period defined as metamorphosis. We prefer calling metamorphosis the continuous, smooth, and relatively rapid process that begins when the epidermis of the tornaria thickens, and ends ~ 72 h later when the individual resembles a juvenile worm, which begins to crawl on the benthos (Table 1). In addition, we emphasize that many of the morphological changes that are necessary for metamorphosis occur much earlier, during the larval stage itself, and as a result any definition of metamorphosis is debatable. Below, we discuss the morphological changes that occur during metamorphosis in *Schizocardium californicum*. These changes involve the loss of larval-specific structures, the development of adult-specific traits, and the remodeling or reorganization of pre-existing structures.

#### Loss of larval-specific structures of the larval body plan

Similar to previous descriptions, we showed that the larval ciliary bands disappear at metamorphosis. As in other species, the circumoral ciliary band regresses early and rapidly, while the telotroch remains after formation of the proboscis, and only fully disappears days after settlement. The other main larval-specific structure of tornaria larvae is the apical organ. In *B. misakiensis*, it disappears a few days after settlement of the juvenile [30]. Similarly, we have showed that the apical organ is still visible during late metamorphosis in *S. californicum*, becomes less easily distinguishable from the general nervous system of the proboscis as metamorphosis unfolds, and is absent in post-settlement juveniles.

Other characteristics of the larval body plan that disappear at metamorphosis are the large blastocoelar space, which becomes highly reduced as a result of the coelomic expansion, and the thin ectodermal epithelium which is replaced by a thick, complex columnar epithelium, first in the proboscis, and later in the forming collar and trunk. Two major unsolved questions are whether or not cells of the larval ectoderm survive metamorphosis, and the developmental origins of the juvenile ectodermal cells.

In *Schizocardium californicum*, the apical strand disappears in the late tornaria. Loss of the apical strand was only described in one other species, *B. misakiensis*, where it disappears shortly before metamorphosis [29]. Additionally, we report here that the circular muscles of the pharynx disappear during metamorphosis in *Schizocardium californicum*.

#### Development of adult specific traits

Adult-specific traits that appear before or during metamorphosis include the mesocoels and metacoels and their derivatives, the muscles of the collar and trunk, the specific neuroanatomy of the adult, the gill slits, and the tripartite body organization with proboscis, collar and trunk.

In all tornaria larvae, the protocoel appears early in development, while the mesocoels and metacoels appear and grow during later larval stages. After reaching a certain size, they stop growing until onset of metamorphosis. A dramatic enlargement of all coelomic cavities is the first sign of metamorphosis. Here, we have showed that muscle fibers appear in all coeloms in the late tornaria larva. During metamorphosis, these give rise very rapidly to the musculature of the juvenile.

The modifications of the nervous system that we describe here are essentially similar to those described in *B. misakiensis* [30], where serotonergic cell bodies and associated nerve plexus form throughout the future proboscis and collar, and a dorsal nerve cord forms along the forming trunk. Our observations add to previous ones by showing that the serotonergic neurons that are scattered across the future proboscis and collar before metamorphosis originate in the late tornaria larva, long before metamorphosis.

A hallmark of the enteropneust body plan is a pharynx with gill slits [6]. The pharynx is lined dorso-laterally by collagenous gill bars that surround perforations of the endoderm. A dorsal to ventral folding of the gill bar (the tongue bar) divides this perforation, creating the characteristic U-shape of enteropneust gill slits. The sides of gill and tongue bars are lined with lateral cilia that create an outward flow of water [67]. The endodermal gill bars are connected to the external environment by ectodermal gill pores. In *Schizocardium californicum*, endodermal gill bars develop in the pharynx of the late tornaria larva. Before metamorphosis begins, the larval gill bars are structurally similar to those of the juvenile, only smaller. Unlike the adult, the endodermal gill bars of the larva are not connected to the external environment. Even though they possess lateral cilia that can be seen beating, the ectodermal gill pores that are necessary to allow an outward water flow only develop during metamorphosis. As a result, it is difficult to assess whether or not gill bars play any role in the larva. In all other species described to date, gill slits only develop immediately before, or after metamorphosis.

Finally, one of the main features of the hemichordate adult body plan is a body divided into prosome, mesosome and metasome. In enteropneusts, these body regions take the form of a proboscis, collar and trunk. Tracking the various body regions of the tornaria through metamorphosis shows that even though the division of the tornaria into a proboscis, collar and trunk is not as obvious as in the adult, it is still recognizable. In the late tornaria larva, the pre- and postoral loops of the circumoral ciliary band define a region of ectoderm, the oral field, that divides the preoral ectoderm from the rest of the body. This region of the ectoderm becomes the proboscis at metamorphosis. Posterior to the mouth, the postoral loop of the ciliary band becomes the anterior boundary of the collar. The posterior collar boundary is not marked by any visible morphological landmark in the larva, but becomes visible at the beginning of metamorphosis. Importantly, gene expression data shows that the future trunk of the adult is specified in the late tornaria larva [68]. These data suggest that even though the collar-trunk boundary is not obvious from morphology alone in the larva, it is already present at the molecular level.

#### Rearrangement of existing body parts during metamorphosis

Our observations show that metamorphosis not only involves gain and loss of specific structures, but also large-scale changes in their relative size and position. In adult enteropneusts, prosome, mesosome, and metasome define regions of the body plan that include specific components of each germ layer. Our observations show that in the late tornaria larva, endodermal and mesodermal components of all three regions are present, but they do not align with each other and with the ectoderm. Rather, each germ layer is divided into these three regions independently from each other, and only come together at metamorphosis to form the adult proboscis, collar and trunk.

## Conclusion

The embryological, developmental and morphological data described here provide the basis for future studies in *Schizocardium californicum*. Questions that are of interest to molecular developmental studies include axial patterning, induction of germ layers, neurogenesis and neural patterning, the relative contribution of set aside cells and transdifferentiation during metamorphosis, and comparisons with other larval forms and with direct developing enteropneusts. The practical advantages of *Schizocardium californicum* relative to other species, combined with the detailed developmental data presented here, will allow future research to address these questions.

## Abbreviations

A/V: Animal-vegetal
AP: Anteroposterior
dpf: Days post-fertilization
FSW: Filtered sea water
hpf: Hours post-fertilization
PBS: Phosphate buffered saline
RT: Room temperature
